# Precursor prioritization for *p*-cymene production through synergistic integration of biology and chemistry

**DOI:** 10.1186/s13068-022-02226-7

**Published:** 2022-11-17

**Authors:** Hsi-Hsin Lin, Daniel Mendez‐Perez, Jimin Park, Xi Wang, Yan Cheng, Jiajie Huo, Aindrila Mukhopadhyay, Taek Soon Lee, Brent H. Shanks

**Affiliations:** 1 Department of Chemical and Biological Engineering, Iowa State University, Ames, IA 50011 USA; 2 Center for Biorenewable Chemicals (CBiRC), Iowa State University, Ames, IA 50011 USA; 3 Joint BioEnergy Institute, 5885 Hollis Street, Emeryville, CA 94608 USA; 4 Biological Systems & Engineering Division, Lawrence Berkeley National Laboratory, Berkeley, CA 94720 USA; 5 Department of Chemical and Biomolecular Engineering, University of California, Berkeley, CA 94720 USA

**Keywords:** Limonene, 1,8-Cineole, *p*-Cymene, Dehydrogenation, Chemistry/biology integration, Fermentation

## Abstract

The strategy of synergistic application of biological and chemical catalysis is an important approach for efficiently converting renewable biomass into chemicals and fuels. In particular, the method of determining the appropriate intermediate between the two catalytic methods is critical. In this work, we demonstrate *p*-cymene production through the integration of biosynthesis and heterogenous catalysis and show how a preferred biologically derived precursor could be determined. On the biological side, we performed the limonene and 1,8-cineole production through the mevalonate pathway. Titers of 0.605 g/L and a 1.052 g/L were achieved, respectively. This difference is in agreement with the toxicity of these compounds toward the producing microorganisms, which has implications for subsequent development of the microbial platform. On the heterogeneous catalysis side, we performed the reaction with both biological precursors to allow for direct comparison. Using hydrogenation/dehydrogenation metals on supports with acid sites, both limonene and 1,8-cineole were converted to *p*-cymene with similar yields under equivalent reaction conditions. Thus, we could determine that the most promising strategy would be to target 1,8-cineole, the higher titer and lower toxicity bio-derived precursor with subsequent catalytic conversion to *p*-cymene. We further optimized the biological production of 1,8-cineole via fed-batch fermentation and reached the titer of 4.37 g/L which is the highest known 1,8-cineole titer from microbial production. This work provides a valuable paradigm for early stage considerations to determine the best route for the high-efficiency production of a target biobased molecule using an integration of biology and chemistry.

**Supplementary Information:**

The online version contains supplementary material available at 10.1186/s13068-022-02226-7.

## Introduction

While biosynthesis and chemical catalysis are commonly viewed as alternative strategies for converting biomass-derived feedstocks into chemical products, their differing conversion characteristics can make them complementary [1, 2]. In general, biosynthesis provides a more selective tool for biomass conversion, as demonstrated by its high regioselectivity and use of milder reaction conditions that can further diminish possible side reactions. These advantages are particularly relevant to processing biobased feedstocks that contain multiple functional groups. However, there are also important challenges for biosynthesis, such as operating temperature constraints, product toxicity toward microorganisms, and other factors limiting conversion efficiencies [3]. In contrast, chemical catalysts can operate in a wide range of reaction conditions and have well-established scaling knowledge to industrial processes [4]. However, the selectivity of heterogeneous catalysts for converting multifunctional compounds can be quite challenging. Their respective advantages suggest the integration of the two conversion methods can provide potential synergies for producing renewable molecules [5–7].

To realize biosynthetic and chemical conversion synergies, identification of the optimal intermediate molecule to use in the integrated biological and chemical process is critical. Typically, studies on intermediate molecules in the literature focus on interesting platform molecules that could yield valuable derivatives [8–10]. In contrast, there are fewer studies that start from the target product molecule and work backward to determine the more promising biological intermediate if multiple options exist [11]. The optimal integration of biosynthesis and chemical catalysis should consider the efficiency and challenges of both parts of conversion and explore possible integration workflows via different intermediates. In this work, the production of biobased *p*-cymene is demonstrated as an example to illustrate this concept.

*p*-Cymene is a versatile target renewable molecule as it can be used as a fuel [12] or be converted into value-added products, such as cuminaldehyde, [13] cumic acid [14], 4-hydroxybenzoic acid, [15] and terephthalic acid [16]. In general, p-cymene has received attention as researchers have proposed methods for producing it via catalytic alkylation of toluene, but selective control of the reaction is still a defining issue [17, 18]. Given the range of end products accessible and the non-renewable aspect of producing it directly from petrochemicals, there is motivation to develop a selective transformation to renewable biomass-derived p-cymene.

One approach is utilizing the terpenoid biosynthetic pathway to generate limonene that can be dehydrogenated to *p*-cymene. Limonene, a natural product, can be synthesized through cyclization of geranyl pyrophosphate (GPP) produced from either the methylerythritol 4-phosphate (MEP) pathway or the mevalonate (MVA) pathway by limonene synthase [19]. Several studies have demonstrated limonene production in a microbial system [20]. The steps to convert limonene to valuable derivatives, including the dehydrogenated product, *p*-cymene, have also been explored [21–23].

Alternatively, the same terpenoid pathway can be used to generate 1,8-cineole using 1,8-cineole synthase instead of limonene synthase. A biosynthetic route to 1,8-cineole was reported recently in *E. coli* [3]. Given the molecular structure of 1,8-cineole, it could potentially serve as an alternative intermediate to *p*-cymene. Converting limonene or 1,8-cineole to *p*-cymene through a biosynthetic pathway requires additional steps, which have not been experimentally well-developed and could prove challenging [24]. Given the uncertainty of *p*-cymene synthesis exclusively through biosynthesis, finding an appropriate hand-off point from biological intermediate to chemical synthesis provides an advantaged alternative route.

Applying limonene dehydrogenation to produce *p*-cymene has been widely reported with both metal and metal oxide catalysts with supported palladium as the most commonly used [25–28]. Lesage et al. performed dehydrogenation of limonene on a Pd/SiO_2_ catalyst [29]. They further improved the product yields by adding extra alkene as a hydrogen acceptor. Weyrich et al. proposed that cerium promotion of Pd supported on ZSM-5 achieved a higher *p*-cymene selectivity and slower catalyst deactivation [30]. Garu et al. examined the initial rates of isomerization, hydrogenation, and dehydrogenation in the presence of different Pd loadings on Al_2_O_3_ or carbon [31]. Buhl et al. also studied Pd/SiO_2_ in the reaction, as well as examining the effect of other supports on the catalytic performance [32, 33]. Using Pd nanoparticle catalysts in the aqueous phase, Zhao et al. investigated the effects of temperature, H_2_ pressure, and the catalyst loading [34]. In addition to catalyst studies, researchers have explored reactor-level strategies for limonene dehydrogenation to *p*-cymene. Zhang et al. developed a sprayer system with a Pd/C catalyst that gave the potential for improved reaction performance at a larger scale [35]. Zhang and Zhao proposed an integrated reaction system in which the hydrogen produced via the Pd-catalyzed dehydrogenation could be directly and simultaneously used in the same batch reactor in bio-jet production through hydrodeoxygenation of triglycerides or deoxygenation of fatty acid to produce C_14_–C_18_ alkanes [36, 37]. More recently, Yılmazoğlu and Akgün compared the reaction performance of Ni, Pt, and Pd supported on Al_2_O_3_ in supercritical conditions with ethanol and 2-propanol. An 80% yield of *p*-cymene has been reached with Pd/Al_2_O_3_ [38]. As shown in these examples, limonene can be converted to *p*-cymene with high conversion and selectivity in fixed-bed reactors or coupled with other reactions in batch reactors as a proton source.

1,8-Cineole conversion to *p*-cymene has received less attention, but the reaction has also been reported. Benjamin et al. converted 1,8-cineole to *p*-cymene utilizing Mo, Cr, Fe, Co, or Pd supported on γ-Al_2_O_3_ [39]. Pd was found to be most effective for the transformation, and they further performed a kinetic study with it [40]. Therefore, these previous studies demonstrated that either limonene or 1,8-cineole could be used as a biological-derived reactant for synthesizing *p*-cymene. However, there has been no direct comparison of the overall efficiency of these competing possibilities, which is critical for selecting the preferred reaction route for further development.

**Fig. 1:**
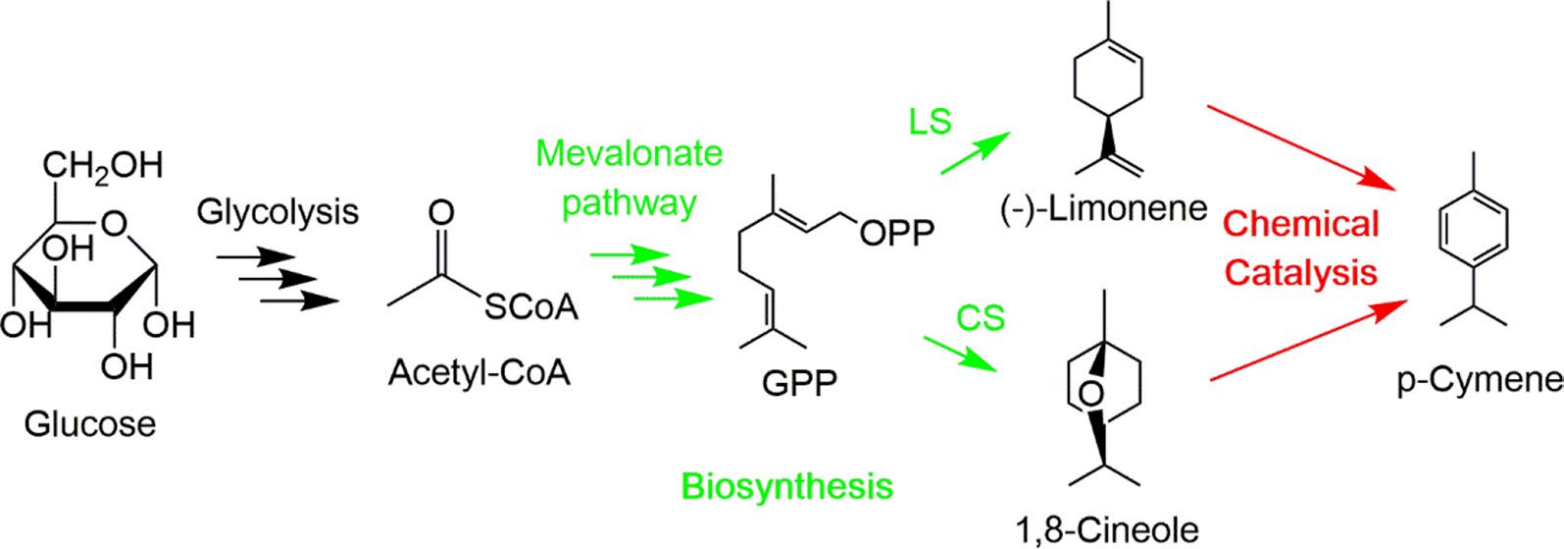
Production of *p*-cymene through biological production of monoterpenes and chemical catalysis. (LS, limonene synthase; CS, 1,8-cineole synthase)

In this work, we examined the holistic conversion of glucose to *p*-cymene through two different routes using the integration of biological and chemical conversion: (a) glucose to limonene to *p*-cymene and (b) glucose to 1,8-cineole to *p*-cymene. The proposed reaction pathways are shown in Fig. 1. The goal of this work is to provide an overall comparison of the pathways, such that a decision can be made as to the most promising biological intermediate target, which could then be chemically converted to *p*-cymene. By identifying the preferred reaction pathway, guidance can be provided on subsequent work for the microbial system and chemical catalyst optimization as well as process integration. Overall, this work provides a model of how to use early stage considerations to evaluate intermediate molecules for the high-efficiency production of a target biobased molecule.

## Results and discussion

### Biological production of the monoterpene intermediates

We have reported previously that the monoterpene pathway can be engineered for microbial production of both limonene and 1,8-cineole in *E. coli*.[3, 41] By heterologous expression of the MVA pathway, engineered *E. coli* strains were able to produce about 600 mg/L and 650 mg/L of limonene and 1,8-cineole, respectively, from 1% glucose as the carbon source. Based on this previous work, the microbial system was further optimized in this study to produce limonene and 1,8-cineole as the intermediates toward *p*-cymene conversion (Fig. 1). For limonene production, a modified 2-plasmid system was constructed with the heterologous MVA pathway [42]. *E. coli* DH1 strain harbouring the MVA pathway plasmids JPUB_017011 and JPUB_017013 (Table 1) produced 605 mg/L from 1% glucose after 72 h, which was comparable to the highest production levels reported to date for the limonene producing systems [43].

**Table 1:** Strains and plasmids used in this study

Strains	Description	Reference
2pLimA	*E. coli* DH1 with plasmids JPUB_017011 + JPUB_017013	[42]
DM01 (JBEI-15051)	*E. coli* DH1 with S81F mutation in *ispA*	[3]
DM02 (JBx_136491)	DM01 with plasmids JBEI-15240 + JBEI-15060	[3]
DM03 (JBEI-15050)	*E. coli* MG1655 with mutation (S81F) in *ispA*	This study
DM04 (JBx_136471)	DM03 with plasmids JBEI-15240 + JBEI-15060	This study
DM05 (JBx_136467)	DM01 with deletions *ΔpoxB Δpta ΔackA*	This study
DM06 (JBx_136472)	DM03 with deletions *ΔpoxB Δpta ΔackA*	This study
DM07 (JBx_136468)	DM05 with plasmids JBEI-15240 + JBEI-15060	This study
DM08 (JBx_136470)	DM06 with plasmids JBEI-15240 + JBEI-15060	This study
DM09 (JBx_136473)	DM06 with deletion *ΔldhA*	This study
DM10 (JBx_136474)	DM09 with plasmids JBEI-15240 + JBEI-15060	This study
DM11 (JBx_137160)	DM09 with plasmid JBx_136481 + JBEI-15060	This study
DM12 (JBx_137158)	DM09 with plasmids JBEI-15240 + JBx_136482	This study
DM13 (JBx_137159)	DM09 with plasmids JBx_136481 + JBx_136482	This study

Plasmids	Description	Reference
JPUB_017011	pBbA5a-MTSA-T1-MBI; 7 genes of the MVA pathway (from thiolase to IPP isomerase) for IPP and DMAPP production	[42]
JPUB_017013	pBbE1k-trGPPS-LS; truncated GPP synthase and limonene synthase under Trc promoter	[42]
JBEI-15240	pBbA5c-MTSA-T1-MBI-T1002-Ptrc-CS_Str_; JPUB_17011 with bacterial cineole synthase added under Trc promoter	[3]
JBEI-15060	pTrc99a-GPPS-CS_Str_-ispA*; GPP synthase and bacterial cineole synthase under Trc promoter	[3]
JBx_136481	pBbA5c-MTDa-T1-MBI-T1002-Ptrc-CS_Str_	This study
JBx_136482	pTrc99a-GPPS-CS-ispA(S81F)-HMGR_Da	This study

**Fig. 2:**
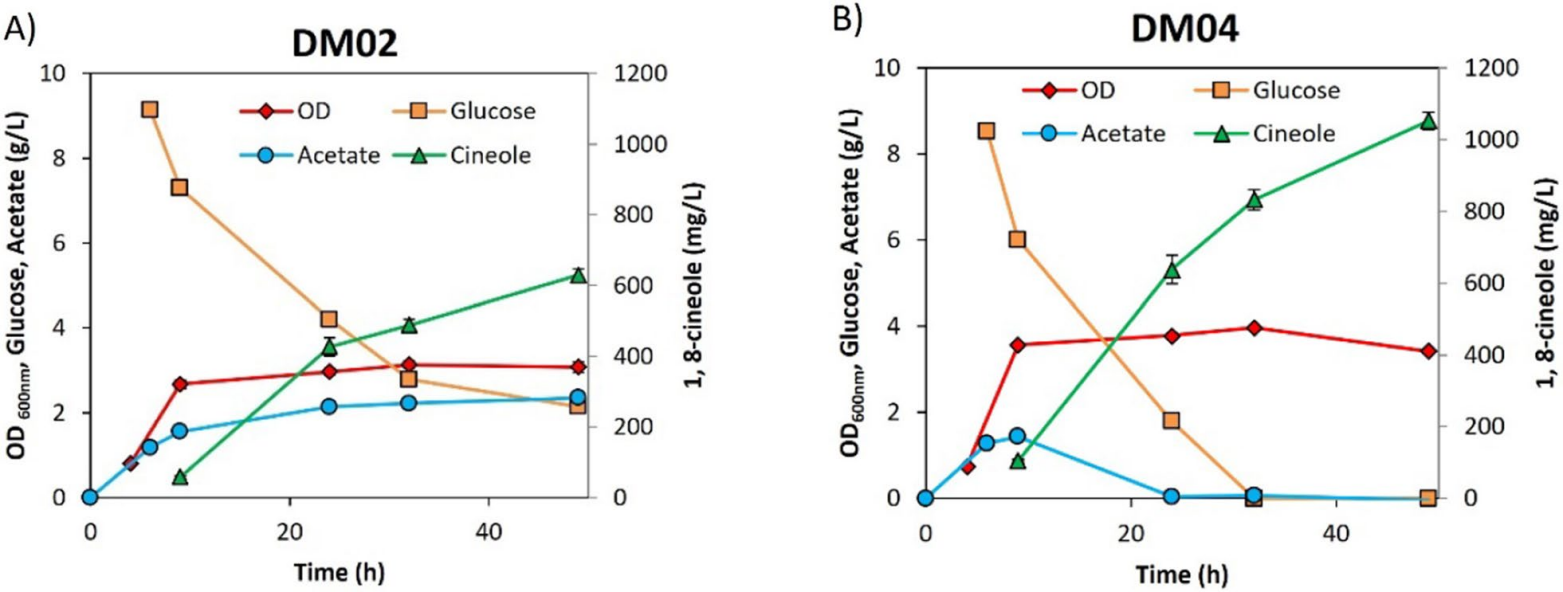
Comparison of 1,8-cineole production from strains DM02 (*E. coli* DH1 background) and DM04 (*E. coli* MG1655 background). **A** 1,8-cineole production from the strain DM02. **B** 1,8-cineole production from the strain DM04. Production experiment was performed in test tubes (triplicates) using EZ rich defined medium supplemented with 1% glucose at 30 °C. Cultures were induced at OD_600nm_ 0.8 with 500 µM IPTG, a 10% dodecane overlay was used for all cultures

Production of 1,8-cineole was originally accomplished using *E. coli* DH1, a strain that has been frequently used for isoprenoid production with the heterologous MVA pathway [3, 41, 44, 45]. As *E. coli* MG1655, a K-12 derived strain that is close to the wild type with minimal genetic manipulation, is often used in metabolic engineering applications at large scales, it was determined whether similar 1,8-cineole production levels could be achieved using *E. coli* MG1655. A mutation in the *ispA* gene (S81F) introduced into the genome of the strain MG1655 has been previously shown to improve GPP pool for monoterpene production (resulting in strain DM03, Table 1), and the two plasmids (JBEI-15240 and JBEI-15060, Table 1) were transformed into this strain resulting in strain DM04 (Table 1). Correspondingly, 1,8-cineole production in the strain DM04 was significantly improved reaching 1052 mg/L after 48 h (Fig. 2), which is more than a 60% increase compared to the production by the *E. coli* DH1 strain (DM02) [3].

### Toxicity comparison of limonene and 1,8-cineole

**Fig. 3:**
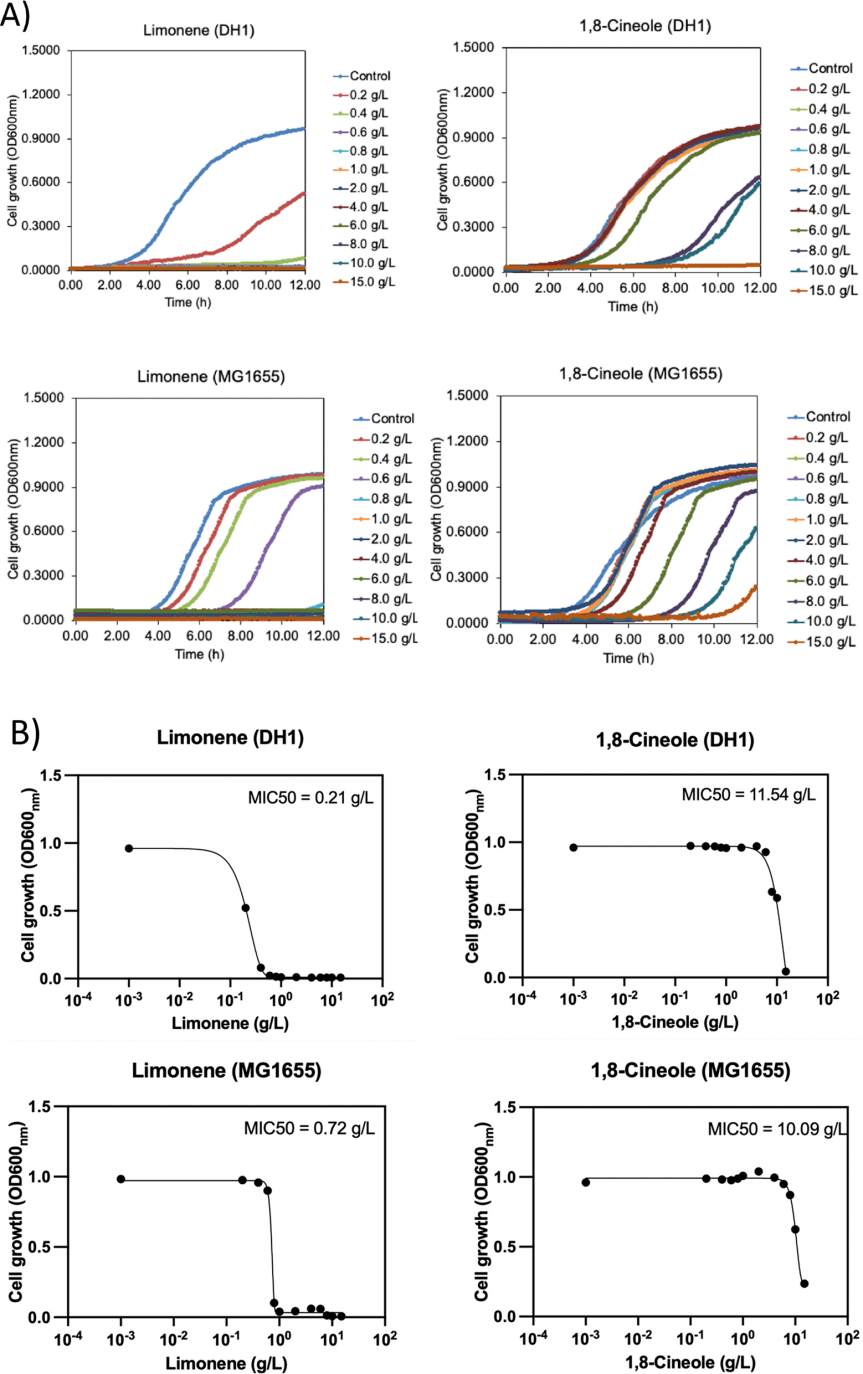
Monoterpene tolerance of *E. coli*. (A) cell growth of *E. coli* strains with different concentrations of monoterpenes; (B) MIC50 of limonene and 1,8-cineole for *E. coli* DH1 and MG1655 strains after 12 h

In microbial production of monoterpenes, limonene has been reported to be toxic to microbial cell growth, while there is less discussion regarding 1,8-cineole toxicity [46]. The different toxicity of monoterpene molecules to the production host may affect their production level during biological production. Given that the production titer of 1,8-cineole was significantly higher than that of limonene from our producing strains, the tolerance of two *E. coli* strains was investigated toward limonene and 1,8-cineole to quantitatively reveal their toxicities on microbial cell growth. As shown in Fig. 3, cell growth of *E. coli* DH1 and MG1655 strains was significantly inhibited in the presence of limonene at 0.6 g/L and 1.0 g/L, respectively, whereas no growth inhibition was observed by 1,8-cineole at the same concentrations. Cell growth showed clear inhibition when 1,8-cineole concentration reached 15 g/L for both *E. coli* DH1 and MG1655 strains, which was 15-fold higher than when limonene was present, but still with less growth inhibition. The minimum inhibitory concentration (MIC50) of limonene and 1,8-cineole showed significant difference. The limonene MIC50 was 0.21 g/L and 0.72 g/L, respectively, for the *E. coli* DH1 and MG1655 strains, whereas the 1,8-cineole MIC50 was 11.54 g/L and 10.09 g/L, respectively, for the two *E. coli* strains (Fig. 3B). Thus, we determined that limonene is more toxic than 1,8-cineole toward *E. coli* cell growth. This result suggests 1,8-cineole could reach higher production level during microbial production, indicating a potentially more favourable route for coupling with downstream chemical conversion. Next, evaluation of the subsequent competing chemical catalytic reaction steps shown in Fig. 1 for the two biological intermediates was performed to compare the overall reaction pathways to *p*-cymene.

### Catalyst physical properties

To directly compare the *p*-cymene production from the two biobased intermediates, catalysts were prepared and used under the same reaction conditions for both intermediates. The synthesized chemical catalysts were characterized, and the physical properties of the resulting materials used in the study are given in Table 2. The surface area of the metal-loaded Al_2_O_3_ supports were from 127.1 to 148.4 m^2^/g, which were similar to the 146.9 m^2^/g of the original Al_2_O_3_. Upon metal impregnation, the pore volume of the catalysts decreased slightly as did the average pore diameter. These minor changes in physical properties would not be expected to significantly impact accessibility of the active metal sites. The surface area of Pd on the SiAl and SiO_2_ supports were 90.8 and 306.3 m^2^/g, respectively. The nitrogen physisorption isotherms are shown in Additional file 1: Figures S1–S7. The metallic surface area of the catalysts was measured using both H_2_ and CO chemical adsorption and are listed in Table 2. Since the metallic surface area of the Cu-based catalyst could not be determined by H_2_ or CO adsorption, it was not measured.

**Table 2:** Physical properties of fresh catalysts

Catalyst	Surface Area (m^2^·g^−1^)	Pore volume (cm^3^·g^−1^)	Average pore width (nm)	Metallic surface area (μmol·g^−1^)	
				H_2_	CO
Pd/SiAl	91	0.384	20.2	22.8	20.2
Pd/Al_2_O_3_	142	0.453	12.4	26.8	32.2
Pd/SiO_2_	306	0.962	11.6	25.9	14.3
Al_2_O_3_	147	0.557	14.5	–	–
Ni/Al_2_O_3_	127	0.438	14.0	–^a^	31.9
Cu/Al_2_O_3_	148	0.512	13.5	–^a^	–^a^
Pt/Al_2_O_3_	144	0.473	13.1	33.3	60.7

^a^not determined

### Catalytic performance of limonene and 1,8-cineole conversion with Pd-based catalysts

**Fig. 4:**
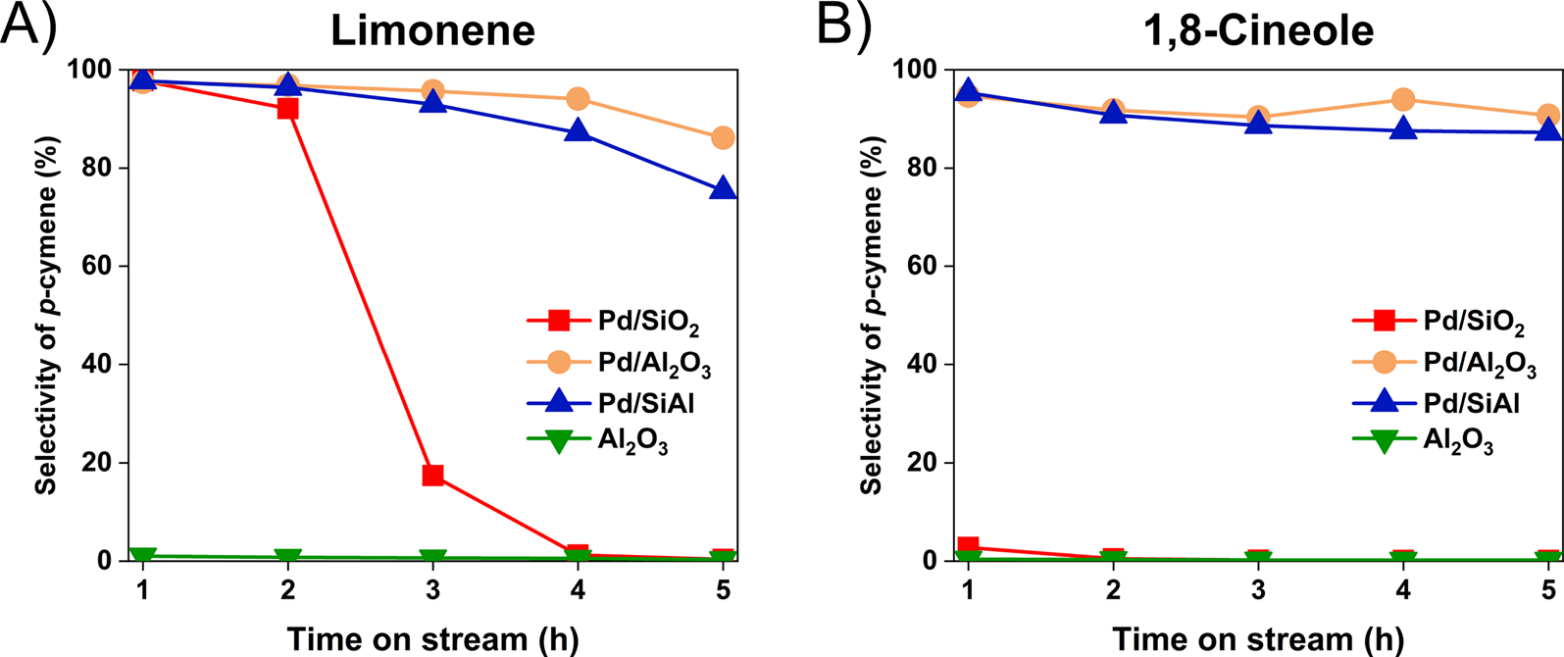
Palladium catalytic performance with (**A**) limonene (**B**) 1,8-cineole as the reactants at 250 °C with 5 wt% of Pd on supports

As noted in Fig. 1, either the limonene or 1,8-cineole pathway can be utilized to generate an intermediate for biobased *p*-cymene synthesis. To study which intermediate is more efficacious for biobased *p*-cymene production, the two reactants were compared using the same catalysts and reaction conditions in a gas-phase fixed bed reactor. Since Pd has been reported to be a promising candidate in both limonene and 1,8-cineole conversion [29, 39], the reaction was first conducted in the presence of 5% Pd on SiAl, γ-Al_2_O_3_, and SiO_2_. An additional trial was conducted with just γ-Al_2_O_3_ to demonstrate the reactivity of the support in the absence of Pd. After the first hour of operation, which included the start-up transient, the mass recovery of the reactant and products was between 91.4 to 96.8 wt% for the subsequent time points. As shown in Fig. 4A, both Pd/SiAl and Pd/Al_2_O_3_ converted limonene to *p*-cymene effectively. The *p*-cymene mass ratio in the products was determined to be between 93.0 and 97.0 wt% until the third hour. Following that time, the yield of *p*-cymene started decreasing, as hydrogenated products and unreacted limonene were observed. By the end of the 5-h run, the *p*-cymene product decreased to 86.2 and 75.3 wt% for Pd/Al_2_O_3_ and Pd/SiAl, respectively. In contrast, the Pd/SiO_2_ showed significant deactivation in converting limonene to *p*-cymene. Only 17.4 wt% of the limonene was converted to *p*-cymene by the third hour. Almost no reactivity was observed after the fourth hour with limonene as the primary compound in the condenser, and the results for γ-Al_2_O_3_ without Pd showed no *p*-cymene product.

The results for the reaction of 1,8-cineole at the same conditions are shown in Fig. 4B. Similar to the limonene reactions, Pd/Al_2_O_3_ and Pd/SiAl showed high reactivity for 1,8-cineole conversion. Initially, the *p*-cymene product yields were 94.7 and 95.3 wt%, respectively, for these catalysts. The amount of the *p*-cymene product gradually decreased to 90.7 and 87.3 wt% over the 5 h run, which was less decrease than observed when limonene was the starting reactant. The additional dehydration reaction required for 1,8-cineole was readily achieved in the presence of the acidic supports without additional process steps, which demonstrated *p*-cymene could be efficiently produced from 1,8-cineole rather than limonene at similar reaction conditions with appropriate catalyst selection.

**Fig. 5:**
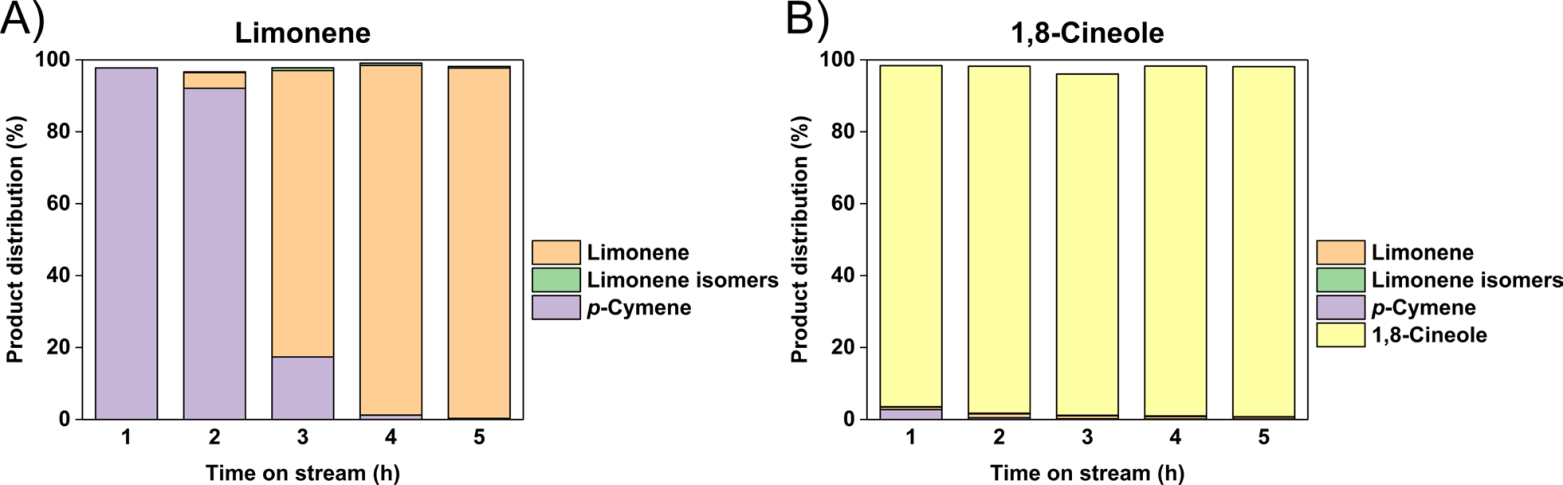
Pd/SiO_2_ catalytic performance with (**A**) limonene (**B**) 1,8-cineole as the reactants

A significant difference in yield was observed when Pd/SiO_2_ was used as the catalyst for the reactants (GC–MS product spectra given in Additional file 1: Figures S11 and S12), so breakdowns of the respective product streams are shown in Fig. 5. As noted from Fig. 4, the yield of *p*-cymene from limonene using Pd/SiO_2_ decreased with time. Figure 5A shows that once the catalyst deactivated, the primary product collected was just limonene and not any other isomers. On the other hand, the initial conversion of 1,8-cineole was only 2.7 wt% and the primary molecule in the effluent (Fig. 5B) was still 1,8-cineole, showing that the dehydration did not occur. Therefore, any weak acid silanol groups on the SiO_2_ were not sufficiently acidic to perform dehydration in contrast to the SiAl or Al_2_O_3_ supports, so the oxygen ring remained primarily unreacted. While unable to produce *p*-cymene either, γ-Al_2_O_3_ alone generated products that were significantly different from Pd/SiO_2_.

**Fig. 6:**
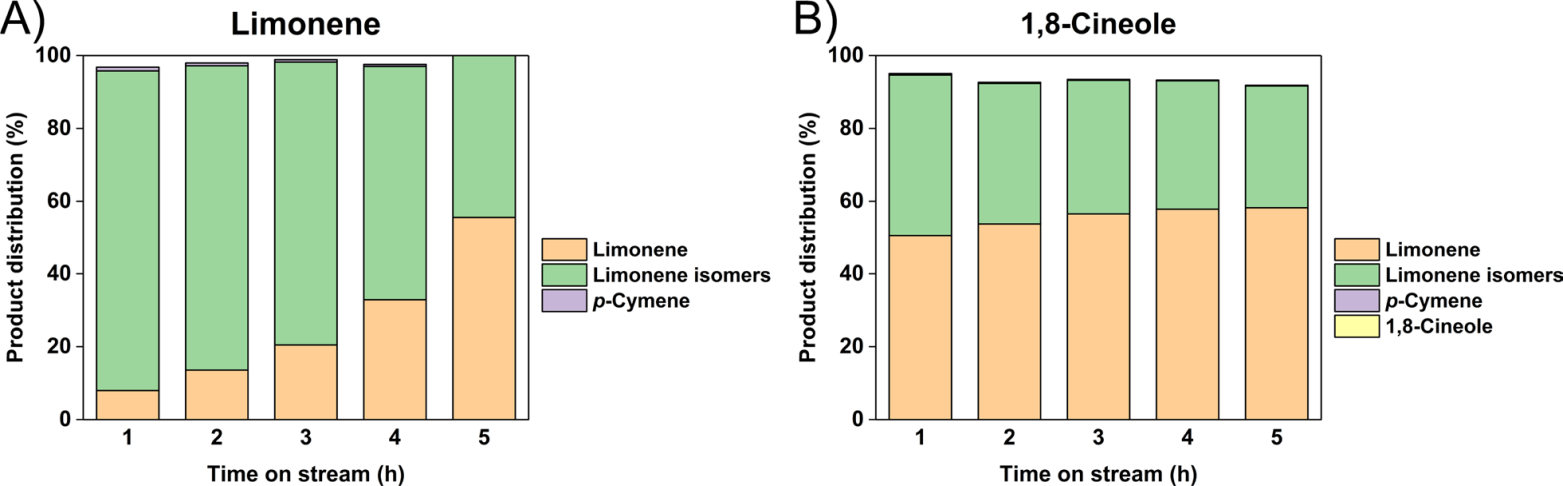
γ-Al_2_O_3_ catalytic performance with (**A**) limonene (**B**) 1,8-cineole as the reactants

The catalytic performance results for just γ-Al_2_O_3_ are shown in Fig. 6 (GC–MS product spectra given in Additional file 1: Figures S13 and S14). Significant isomerization of the limonene reactant was observed. As shown in Fig. 6A, 44.7 to 87.8 wt% of isomers were determined in the products, which suggested that the limonene structure can rearrange under reaction conditions in the presence of γ-Al_2_O_3_. As for the 1,8-cineole run in Fig. 6B, the major products were also limonene and isomers of limonene, which suggested that the acid sites on γ-Al_2_O_3_ were able to react with the oxygen ring structure and convert 1,8-cineole into terpenes. For limonene (Fig. 6A, the isomerization activity gradually decreased, which suggested that some acid site activity loss over time. In contrast, the isomerization activity remained more constant with the 1,8-cineole feed, which suggested that water generated from the additional dehydration step might have helped maintain the acidic sites. Overall, neither of the reactants were converted to *p*-cymene without having Pd on the support, which validated the metallic function was required for hydrogenation/dehydrogenation.

### Other hydrogenation/dehydrogenation metal catalysts

**Fig. 7:**
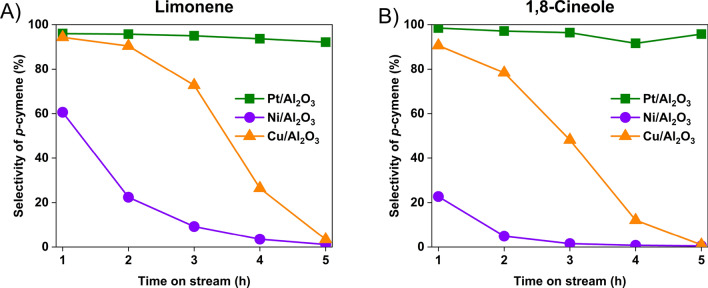
Catalytic performance of 5 wt% metal loading on γ-Al_2_O_3_ with (**A**) limonene (**B**) 1,8-cineole as the reactants under 250 °C

Pd on an acidic support was found to have the dehydration and dehydrogenation activity required to convert either limonene or 1,8-cineole to the target *p*-cymene at the same reaction conditions. In particular, γ-Al_2_O_3_ was an effective acidic support, so further work was performed to determine if other known hydrogenation/dehydrogenation metals could be used in conjunction with γ-Al_2_O_3_ to form an effective catalyst. 5 wt% platinum, nickel, and copper on γ-Al_2_O_3_ catalysts were prepared through the same incipient wetness impregnation method used for Pd. The reaction performance of the resulting catalysts is summarized in Fig. 7.

The Cu/Al_2_O_3_ and Ni/Al_2_O_3_ catalysts showed some reactivity for both limonene and 1,8-cineole conversion with the Cu/Al_2_O_3_ having superior activity to the Ni/Al_2_O_3_. However, neither of the catalysts gave stable performance for either reactant, as the catalysts showed continuous deactivation for increasing time on stream. The nickel-based catalyst showed a particularly noticeable difference between limonene and 1,8-cineole in both its reactivity and stability. The difference could be due the water generated in the dehydration process causing significant deactivation of metallic nickel. A similar stability trend that Pt > Cu > Ni on Al_2_O_3_ was determined in another hydrogen production reaction that involved water [47]. However, the conditions in that study were quite different than the current reaction system, so the results are just suggestive of a possible effect. More study on the spent catalyst surface is required to understand the deactivation mechanism.

The supported Pt catalyst showed stable performance when either limonene or 1,8-cineole was used as the reactant. The amount of *p*-cymene in the products remained around 95 wt% for the entire 5 h test. As shown in Table 2, the incipient wetness synthesis used in this study resulted in a higher number of Pt sites than with Pd despite comparable mass loading of the metal. Therefore, a direct comparison of the intrinsic reactivity of the Pt and Pd sites for these reactions cannot be made. Based on the equivalent weight loading of the four metals, the Pt and Pd were more active for the reaction, which is consistent with general dehydrogenation activity for these four metals. In addition, the higher reactivities for the Pd/Al_2_O_3_ and Pt/Al_2_O_3_ catalysts could be manifest in the apparently better stability as there could be excess active sites in the reactor, which means that even if some were deactivated, the apparent overall activity would remain high. It was not the goal of this study to optimize the catalyst. However, it is clear that further optimization could be achieved, such as lower loading of either the Pd or Pt and improved dispersion of the metals.

Taken together, the reaction results (Figs. 4 and 7) demonstrated that the hydrogenation/dehydrogenation metals tested (Pd, Pt, Ni, Cu) were able to catalyze the dehydrogenation of limonene and its isomers. When coupled with supports, which have acidic sites to catalyze dehydration, both limonene and 1,8-cineole can be readily converted to *p*-cymene. While there might be a slight difference in the stability for Pt and Pd supported catalysts, the extra dehydration step required when 1,8-cineole is the reactant could be readily accomplished with a bifunctional catalyst used under the same reaction conditions as needed to convert limonene.

### Dehydrogenation in dodecane solution

As mentioned in the biosynthesis section, dodecane can be used to extract either the limonene or 1,8-cineole in situ from the fermentation broth as well as to reduce evaporation loss of the biosynthetic products so as to further enhance the product titer. Ideally, this extraction stream should be used with minimal purification in the catalytic step for producing *p*-cymene. Thus as part of the whole integrated reaction pathway screening it was useful to perform an initial assessment whether any difference existed in the conversion of limonene or 1,8-cineole in dodecane.

**Fig. 8:**
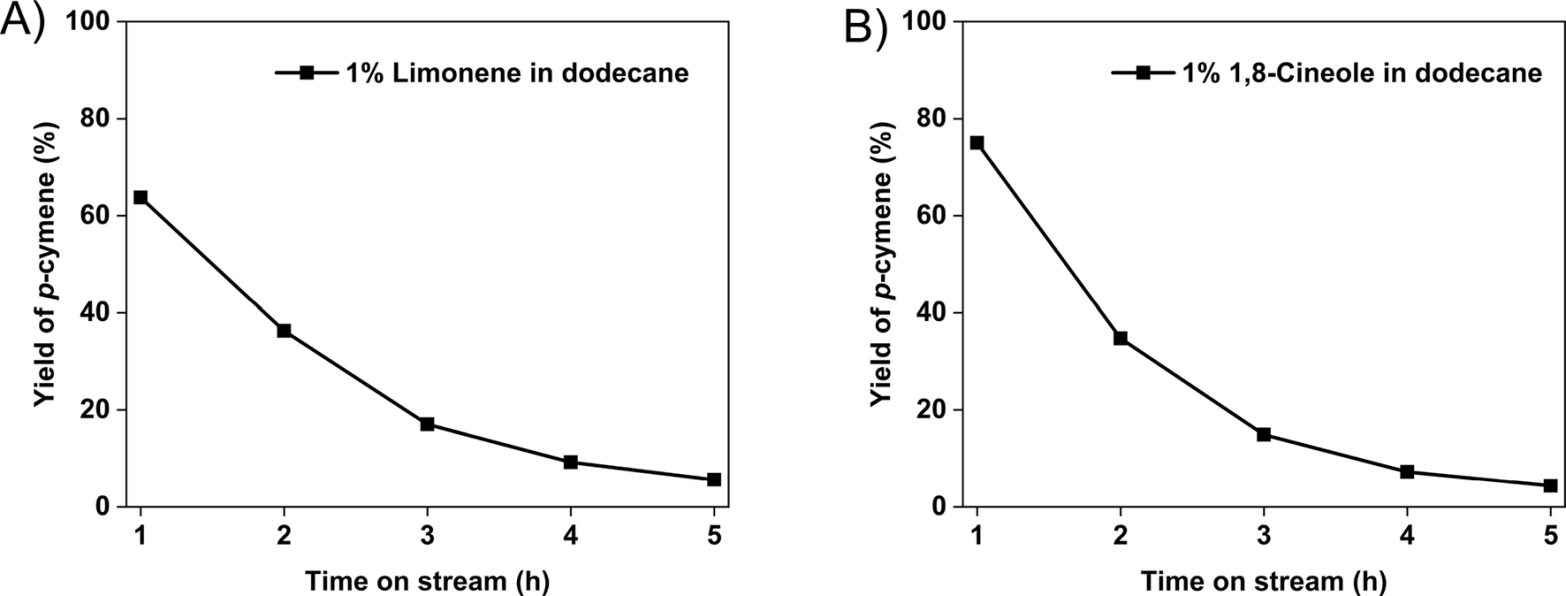
Yield of *p*-cymene with 1.0% (v/v) (**A**) limonene (**B**) 1,8-cineole in dodecane as the reactant

For this study, a 1.0%(v/v) of limonene or 1,8-cineole in dodecane solution was used as the feed. The feed rate of the solution was controlled at 1.0 mL/h and evaporated in a preheater with a 20 mL/min He flow. A 300 mg 5 wt% Pd/Al_2_O_3_ catalyst was packed in the catalytic bed and reactions were controlled under 300 °C. The results are shown in Fig. 8.

Both limonene and 1,8-cineole were similarly converted to *p*-cymene in the presence of the large amount of dodecane, as shown in Fig. 8. As the goal of the test was to validate the reactivity of the respective reactants in dodecane, the amount of catalyst used was not optimized to get full conversion. Unfortunately, the Pd/Al_2_O_3_ catalyst was found to deactivate in both cases, so a more in-depth study of the deactivation mechanism as well as process optimization is required as the next step in process development. As the focus of this work is to determine the preferred target molecule for organism development, the integrated results in the simulated dodecane extraction stream validate that 1,8-cineole rather than limonene is the preferred intermediate target in *p*-cymene production.

### Strain optimization and production of 1,8-cineole in small scale batch cultures

**Fig. 9:**
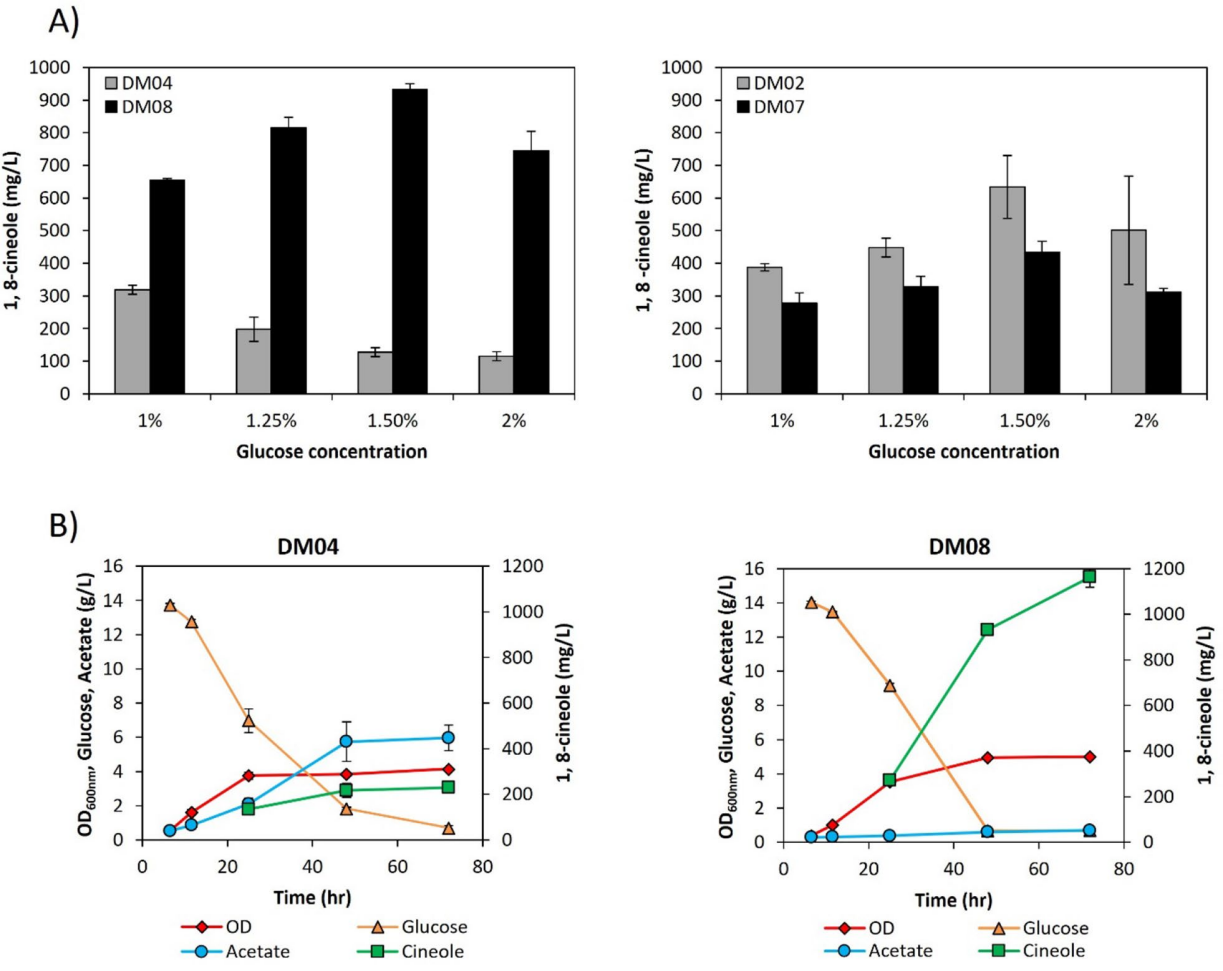
Production in fermentation media. **A** 1,8-cineole production comparison from strains with (DM07 and DM08) and without (DM02 and DM04) the acetate pathways deleted from different glucose concentrations; experiments were run in test tubes using 8 mL of the fermentation medium. **B** 1,8-cineole production time course; 50 mL cultures were grown in shake flasks using the fermentation media with 1.5% glucose. Cultures were inoculated to an OD_600nm_ of 0.1 from an overnight culture, grown at 37 °C and induced at an OD_600nm_ of 1.2 with 1 mM isopropyl β-D-1-thiogalactopyranoside, at the time of induction a 10% dodecane overlay was added and the temperature was changed to 30 °C. All experiments were run in triplicates and the error bars represent the standard deviation

As 1,8-cineole shows a clear advantage as the biosynthetic precursor to *p*-cymene, the production strain was further engineered to improve 1,8-cineole titers. Initial production from strain DM04 already produced over 60% more 1,8-cineole compared to strain DM02 (see Fig. 2). Glucose consumption and fermentation products (acetate, lactate, succinate, formate) were also analyzed. Glucose was fully consumed in strain DM04 at 32 h, but accumulation of acetate was observed for strain DM02, where more than 2 g/L glucose remained in the medium after 48 h. In strain DM02, the acetate level reached 2.3 g/L after 24 h, while there was only an initial accumulation of acetate which disappeared after 24 h for strain DM04 and no other fermentation product was detected at significant levels. It has been reported that production of geraniol (another monoterpene synthesized via the mevalonate pathway) in *E. coli* can be improved by feeding of acetic acid [48]. It has been shown that acetate can be assimilated through the action of the reversible phosphotransacetylase/acetate kinase (Pta-AckA) pathway even in the presence of excess glucose for strain MG1655, resulting in acetyl-CoA accumulation from the consumption of glucose and acetate simultaneously [49, 50]. Since acetyl-CoA is the precursor for the mevalonate pathway, we hypothesized that higher 1,8-cineole levels observed in strain DM04 could be the result of differences in glucose and acetate utilization between the strains at the conditions tested. In these experiments, production of 1,8-cineole was tested using an EZ-rich medium, which is not commonly used for production of commodity chemicals at large scales. Therefore, production using a fermentation medium (defined medium supplemented with glucose and yeast extract, see methods section for detailed composition) was also tested that has been successfully used for production of isoprenol in a 2 L bioreactor [51]. However, 1,8-cineole production for strain DM04 was considerably lower using this fermentation medium only reaching 193 mg/L, which is a fivefold decrease compared to the titer observed using EZ rich medium. To address this, we evaluated the carbon to nitrogen (C/N) ratio differences in the two media. Initially, the concentration of NH_4_Cl in the fermentation medium was 9 mM (C/N of 20), so different NH_4_Cl concentrations were used (C/N ratios from 2.5 to 41.5) to test if nitrogen was limiting; 1,8-cineole production was improved reaching a maximum of 320 mg/L when the C/N was 10 (Additional file 1: Figure S16). We also examined other production parameters. The effect of different induction OD_600nm_ (0.4, 0.8 and 1.2) and IPTG concentrations (IPTG 0.125, 0.5, 1 mM) were tested and the production level improved to 382 mg/L when the cultures were induced at an OD_600nm_ of 1.2 with 1 mM IPTG (Additional file 1: Figure S16) so these conditions were used for the rest of the experimental work (fermentation medium with C/N = 10, induction at OD_600nm_ of 1.2 with 1 mM IPTG). Different glucose concentrations were also tested (1%, 1.25%, 1.5% and 2%) but 1,8-cineole titer decreased at higher glucose concentrations for strain DM04 (Fig. 9A). Interestingly, 1,8-cineole production was higher in this medium for the DM02 strain reaching a maximum of 633 mg/L from 1.5% glucose (Fig. 9B). As can be seen in Fig. 9B, acetate was not assimilated by the DM04 strain under these conditions and accumulated to 5.7 g/L after 48 h, which might explain the lower 1,8-cineole titer compared to rich medium. In the case of strain DM02, acetate also accumulated but only reached a maximum of 2.1 g/L (data not shown). Acetate can be toxic to *E. coli* at concentrations as low as 1 g/L [52, 53] and its biosynthesis is also a significant loss of carbon that can affect production of 1,8-cineole.

To test if 1,8-cineole production could be improved by eliminating acetate production, the two pathways involved in acetate biosynthesis in *E. coli*, acetate kinase/phosphotransacetylase (*ackA/pta*) and pyruvate oxidase (*poxB*), were deleted in both the DM01 and DM03 strains resulting in strains DM05 and DM06, respectively. Plasmids for 1,8-cineole production were transformed into these acetate knockout strains resulting in strains DM07 (DH1 background) and DM08 (MG1655 background) and production was tested at different glucose concentrations (Fig. 9A). There was a significant increase in 1,8-cineole production for strain DM08 compared to DM04 reaching a maximum titer of 932 mg/L from 1.5% glucose (Fig. 9A). Growth was also improved in strain DM08, reaching a maximum OD_600nm_ of 5.0, whereas the maximum OD_600nm_ for strain DM04 was 4.1 (Fig. 9B). Glucose was fully consumed at 48 h for strain DM08, while it took more than 72 h for strain DM04 (Fig. 9B). In the case of the DM07 strain a maximum titer of 435 mg/L was reached from 1.5% glucose, which is lower than the titer observed from strain DM02 (Fig. 9A) suggesting that under these conditions the elimination of acetate was not beneficial for 1,8-cineole production. Further analysis for strain DM07 showed that although acetate did not accumulate, a significant amount of lactate accumulated reaching a maximum of 2.8 g/L at 48 h (data not shown), which might explain the lower titer in this strain. Since the DM08 strain showed the best titers using the fermentation medium, this strain was used for further scale-up experiments in a 2-L bioreactor.

### Fed-batch fermentation for 1,8-cineole production

**Fig. 10:**
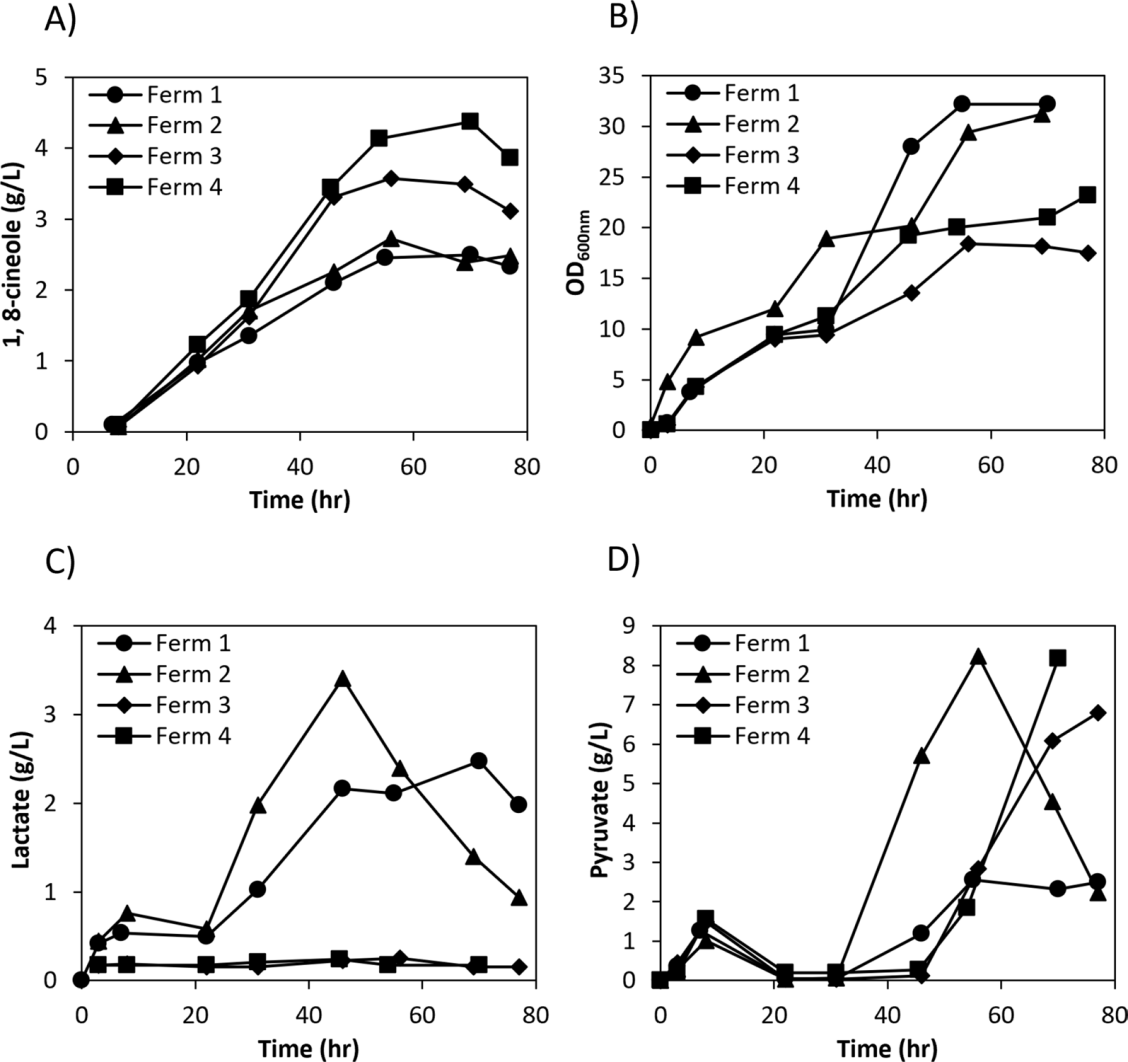
Fed batch fermentation results. **A** 1,8-cineole production; (**B**) Growth comparison; (**C**) Lactate comparison; (**D**) Pyruvate comparison. Fermentations were run in the 2-L bioreactor using the fermentation medium supplemented with 1.5% glucose, feeding started after the glucose was consumed (22–24 h). Ferm 1: strain DM08 (MG1655 background with acetate genes knockout), exponential feeding; Ferm 2: strain DM08, exponential feeding, additional antibiotics (30 mg chloramphenicol and 100 mg carbenicillin) were added at 31 h, Ferm 3: strain DM10 (MG1655 background with acetate and lactate genes knockout), exponential feeding, additional antibiotics (30 mg chloramphenicol and 100 mg carbenicillin) were added at 31 h; Ferm 4: strain DM10, pulse feeding, additional antibiotics (30 mg chloramphenicol and 100 mg carbenicillin) were added at 31 h. The experiments were run in duplicate and the difference was negligible, so the error bars were not presented

The strain DM08 was tested in fed batch fermentations using a 20% dodecane overlay (referred to as Ferm 1 in Fig. 10). Production of 1,8-cineole reached a maximum titer of 2.49 g/L at 70 h with a corresponding OD_600nm_ of 32.2. Although 1,8-cineole production increased linearly during the first 56 h of the fermentation, no significant increase was observed after 56 h suggesting the possibility of plasmid loss during the fermentation. Plasmid instability could be the result of changes in selective pressure due to the inactivation or degradation of the antibiotics over time as well as changes in its concentration (i.e., dilution) due to changes in the fermentation volume during the fed-batch phase. To test if plasmid stability could be improved by increasing the antibiotic selective pressure, additional amounts of both chloramphenicol (30 mg) and carbenicillin (100 mg) were added to the fermenter at 31 h (shown as Ferm 2 in Fig. 10). The additional antibiotics seemed to have a positive effect on plasmid stability, as shown that 1,8 cineole production reached a maximum titer of 2.72 g/L at 56 h, which shows a slightly higher titer compared to Ferm 1. Production of 1,8-cineole in Ferm 2 increased during the first 56 h of the fermentation and decreased after that; analysis of fermentation products (acetate, lactate, succinate, formate) showed that lactate accumulated to significant levels during the fermentation, reaching a maximum of 3.4 g/L at 46 h (Fig. 10C), suggesting that a significant amount of carbon was being diverted to the biosynthesis of this product instead of 1,8-cineole. Like other fermentation products, lactate is produced to achieve redox balance under anaerobic conditions and as a result of overflow metabolism [54]. It has been reported that strains lacking acetate biosynthesis pathways (such as strain DM08) can accumulate significant amounts of lactate and pyruvate [55]. There are three lactate dehydrogenases in *E. coli* that interconvert lactic and pyruvic acids [54], two of them are involved in the conversion of lactate to pyruvate during oxidative growth on lactate, whereas the third one, LdhA, is specific for the production of lactate from pyruvate and has been proposed to have an overspill function [54]. To test if lactate elimination could improve 1,8-cineole production, a mutant strain was constructed in which the *ldhA* gene was knocked out (strain DM09) and the cineole producing plasmids were transformed into this strain, resulting in strain DM10. When this strain was tested in fed-batch fermentations, no significant amount of lactate was produced (Ferm 3 in Fig. 10C) and 1,8-cineole production was substantially increased reaching a maximum titer of 3.57 g/L at 56 h, which is a 31% increase compared to Ferm 2. There was also a substantial accumulation of pyruvate over time for Ferm 2 and Ferm 3 (Fig. 10D), and in both cases there is an initial spike at 8 h. Pyruvate was not detected between 24 and 31 h and then accumulated during the fed-batch phase (feeding starts at 22–24 h) reaching a maximum of 8.2 g/L at 56 h for Ferm 2 and a maximum of 6.7 g/L at 77 h for Ferm 3. Pyruvate accumulation began after 31 h for Ferm 2, whereas in the case of Ferm 3, it began later in the fed-batch phase after 45 h.

Conversion of pyruvate to acetyl-CoA is a key reaction in central metabolism linking glycolysis and the TCA cycle. During aerobic growth the conversion is catalyzed by the pyruvate dehydrogenase (PDH) complex, generating NADH and CO_2_ [56]. The PDH complex is regulated at the transcriptional level by the pyruvate-responsive autoregulator repressing transcription in the absence of pyruvate [57] and at the enzyme level, inhibited by acetyl-CoA and NADH [58]. It has been reported that production of isoprenoids from the mevalonate pathway generates an excess of NADH (conversion of pyruvate to acetyl-CoA) that might result in redox imbalance [59]. Therefore, it is possible that the accumulation of pyruvate in our fermentations could be the result of PDH inhibition due to excess NADH generation. An intermediate step in the mevalonate pathway used in this study (conversion of HMG-CoA to mevalonate) is catalyzed by the NADPH-dependent HMGR from *Staphylococcus aureus*, so the use of an NADH-dependent HMGR instead of NADPH-dependent HMGR has been proposed as a promising strategy to alleviate redox imbalance [60]. To test if pyruvate accumulation could be reduced using this strategy, the NADH-dependent HMGR from *Delftia acidovorans* (HMGR_Da) from different plasmid configurations was expressed and tested for production in batch cultures using test tubes (Additional file 1: Figure S17). When only one copy of the HMGR_Da was expressed from the medium copy plasmid (replacing the NADPH-dependent HMGR, strain DM11) low production was observed, reaching a maximum titer of 137 mg/L (Additional file 1: Figure S17). Two other configurations where the HMGR_Da was expressed from the high copy plasmid in addition to the expression of the *Staphylococcus aureus* HMGR in the medium copy plasmid (strain DM12) or where the HMGR_Da was expressed from both the medium and high copy plasmids (strain DM13) resulted in 1,8-cineole titers of 1.12 g/L and 1.17 g/L, respectively (Additional file 1: Figure S17), which are comparable to the titers observed in strain DM10. Although pyruvate accumulated to similar levels in both strains DM12 and DM13 at 48 h (> 6 g/L), lower levels were observed at 24 and 32 h when two copies of the NADH-dependent HMGR were used (strain DM13 in Additional file 1: Figure S17C) so we decided to test this strain in the bioreactor (Ferm 3_Da, Additional file 1: Figure S18). Pyruvate also accumulated during the fermentation Ferm 3_Da reaching a maximum of 4.8 g/L at 70 h. Although this level is lower than observed in Ferm 3, pyruvate was produced and accumulated over the course of the entire fermentation, whereas in Ferm 3 there was an initial spike at 8 h, not detected between 24 and 31 h, and then accumulates. The 1,8-cineole titer reached a maximum of 1.58 g/L at 46 h for Ferm 3_Da, which is less than half of what was observed for Ferm 3. It is possible that the lower 1,8-cineole titer might be the result of differences in HMGR_Da expression or activity impacting fluxes in the already optimized mevalonate pathway in strain DM10 (also supported by the fact that to reach high titers in strain DM13 the HMGR_Da had to be expressed from both plasmids, whereas only one copy of the NADPH-dependent HMGR expressed from the medium copy plasmid was required for strain DM10) but further strain engineering and optimization would be required to confirm this hypothesis.

Besides pyruvate accumulation, analysis of Ferm 3 showed that glucose consumption was slower compared to Ferm 2 (Additional file 1: Figure S19) resulting in accumulation of glucose during the fed batch phase. To test if accumulation of glucose could be prevented during the fed-batch phase, the feeding strategy was changed from exponential feeding to pulse feeding based on DO (Ferm 4). Briefly, a fixed amount of glucose was added to the tank and the DO of the culture was monitored, additional glucose was only added when a spike in DO was observed. The amount of glucose added was adjusted to maintain the glucose consumption at around 1 g/h (see materials and methods for detailed description). Although pyruvate also accumulated in Ferm 4 at similar levels to those in Ferm 3, the pulse feeding strategy significantly improved 1,8-cineole production reaching a maximum of 4.37 g/L at 70 h which is a 22% increase compared to Ferm 3; the maximum productivity was 0.076 g L^−1^ h^−1^ and the maximum yield was 0.11 g/g glucose, which corresponds to 30% of the maximum stoichiometric yield.

## Conclusion

Biobased *p*-cymene is an interesting target for developing renewable fuels and chemicals. Historically, studies focused on generating *p*-cymene from a limonene intermediate rather than 1,8-cineole, but no direct comparison of the two candidates have been reported. This work provides an early stage comparison of potential reaction pathways that assesses both the biological and catalytic components, and is important for guiding subsequent research and development work for the target molecule. By integrating the experimental results for potential biological and chemical catalytic synthetic steps, 1,8-cineole was found to be a preferred intermediate over the more commonly examined limonene intermediate.

On the biological side, the production of two monoterpenes, limonene and 1,8-cineole, as precursors of *p*-cymene was demonstrated in *E. coli* through engineering of the heterologous isoprenoid biosynthetic pathway. The 1,8-cineole production achieved a 1.05 g/L batch production titer, which is higher than the limonene titer, 0.6 g/L. In addition, the toxicity comparison between the two products showed limonene to be more toxic than 1,8-cineole for *E. coli* cell growth. Taken together, the results suggested that 1,8-cineole would be a more favorable biological intermediate target. Therefore, 1,8-cineole production strains were further engineered, and production parameters were optimized. The best strain (MG1655 derived strain without acetate and lactate pathways) was used for fed-batch fermentations, where 1,8-cineole was produced at 4.37 g/L, which is the highest reported titer for this compound (up to now, the highest titer had been reported in *Rhodosporidium toruloides* at 1.4 g/L [61]); the maximum yield was 0.11 g/g glucose, corresponding to 30% of the maximum stoichiometric yield, and a maximum productivity of 0.076 g L^−1^ h^−1^.

On the chemical side, *p*-cymene synthesis from both candidate intermediates was demonstrated in a fixed-bed gas-phase reactor. While Pd/Al_2_O_3_, Pd/SiAl, and Pd/SiO_2_ catalysts could convert limonene into *p*-cymene, an acidic support was needed for 1,8-cineole conversion to *p*-cymene in a single reaction, so only Pd/Al_2_O_3_ and Pd/SiAl were effective. Other hydrogenation/dehydrogenation metals, including Cu, Ni, and Pt, supported on Al_2_O_3_ were also tested. With either limonene or 1,8-cineole as the reactant, Pt showed the highest reactivity among the three catalysts. All of the testing was performed at the same conditions and any of the supports are easily obtained, so 1,8-cineole was just as easily converted to *p*-cymene as limonene. The additional reaction steps for 1,8-cineole conversion can be simply integrated in the reactor with appropriate catalyst design.

In summary we demonstrated that combining biological and chemical catalysis steps to produce a target molecule requires taking into account aspects of each conversion step, but also the best biological-derived intermediate to handoff to the chemical conversion step. Considering either conversion step in isolation would not necessarily lead to the optimal overall conversion strategy. As organism development is resource intensive, it is useful to develop an early stage comparison to guide selection of the biological intermediate molecule prior to subsequent optimization work.

## Experimental section

### Reagents and materials

Limonene (96%, Acros Organics), 1,8-cineole (98%, TCI America), *p*-cymene (99%, Acros Organics), dodecane (99%, TCI America), chloroplatinic acid hexahydrate (Sigma-Aldrich), palladium (II) nitrate hydrate (Stream Chemicals Inc.), nickel (II) nitrate hexahydrate (Sigma-Aldrich), copper (II) nitrate trihydrate (Sigma-Aldrich), SiO_2_ (Sigma-Aldrich), γ-Al_2_O_3_ (Inframat Advanced Materials).

### Microbial production of monoterpenes

Plasmids and strains used in this study were constructed using standard molecular biology techniques and are described in Table 1 (sequences and plasmid maps are available at https://public-registry.jbei.org). *E. coli* DH1 bearing two plasmids (JPUB_017011 and JPUB_017013) was used for limonene production. Starter cultures were prepared by growing single colonies in LB medium containing 100 µg/mL carbenicillin and 50 µg/mL kanamycin at 37 °C with 200-rpm shaking for overnight. The overnight cultures were diluted in 5 mL EZ-Rich defined medium (Teknova, CA, USA) containing 10 g/L glucose (1%, w/v), 100 µg/mL carbenicillin and 12.5 µg/mL kanamycin, and 0.1 mM IPTG in 50-mL test tubes. Dodecane (10%, v/v) was added as the solvent overlay after the induction. The C/N ratios mentioned in the text correspond to the mass of glucose over the mass of NH_4_Cl. The *E. coli* cultures were incubated in rotary shakers (200 rpm) at 30 °C for 72 h.

For 1,8-cineole production, *E. coli* MG1655 (DM04) with the *ispA* mutation for GPP production harboring two plasmids (JBEI-15240 and JBEI-15060) for the MVA pathway and 1,8-cineole synthase was used (Table 1) [3]. Starter cultures were grown in LB medium containing 30 µg/mL chloramphenicol and 100 µg/mL carbenicillin at 37 °C with 200-rpm shaking for overnight. The overnight cultures were diluted in 5 mL EZ-Rich defined medium (Teknova, CA, USA) containing 10 g/L glucose (1%, w/v), 30 µg/mL chloramphenicol and 100 µg/mL carbenicillin, and the culture was induced with 0.5 mM IPTG in 50-mL test tubes. Dodecane (10%, v/v) was added as the solvent overlay after the induction. The *E. coli* cultures were incubated in rotary shakers (200 rpm) at 30 °C for 48 h.

### *E. col*i tolerance test toward monoterpenes

*E. coli* strains were grown in LB medium overnight and diluted into 0.2 mL EZ-Rich defined medium (Teknova, CA, USA) containing 10 g/L glucose (1%, w/v) and appropriate amounts of limonene or 1,8-cineole in a Corning flat bottom 96-well transparent plate. The initial optical density at 600 nm (OD_600_) was set to 0.01. Cell growth in 96-well plates was monitored using an automated reader, shaker, and incubator (Tecan-F200pro) at 30 °C for 12 h. MIC50 (minimum inhibitory concentration required to inhibit the growth of 50% of organisms) was calculated and fitted as described by Lambert et al.[62].

### Catalyst synthesis

The γ-Al_2_O_3_ and SiO_2_ supports were used as received, whereas the SiAl was synthesized for the study. For the SiAl synthesis, 40 g of sodium metasilicate nonahydrate and 23 g aluminum sulfate octadecahydrate were dry mixed in a stirred round bottom flask, then dissolved in 500 mL of water to prepare a support of silica: alumina ratio of 4:1. Once dissolved, the precipitate formed simultaneously, and dilute hydrochloric acid solution was then added dropwise until the pH reached 7, measured by a pH meter. After stirring for 24 h, the precipitate was filtered and washed three times in nano-pure water. The resultant filter cake was dried at 120 °C overnight. Then, the dried support was ion-exchanged five times with a 0.5 M solution of ammonium nitrate at 50 °C for 24 h. The resultant solid was dried at 120 °C, crushed and calcined at 550 °C for 4 h.

The metals were loaded onto the support materials by incipient wetness impregnation. Metal nitrates were dissolved in nanopure water of the desired volume, according to the pore volume of the support. Catalysts after impregnation were dried at 120 °C for 12 h. The dry particles were gradually heated to 120 °C with a 10 °C /min ramp and maintained for 1 h to remove residual moisture. Then, the temperature was further raised to 550 °C and was held for 4 h. After calcination, the fine powder was pressed in a pellet die set to make pellets. The pressed pellets were crushed, and the particles were further sieved to select particles between 250 to 355 μm. The metal catalysts were named M/support, where *M* = Pd, Pt, Ni, and Cu and support = SiAl, Al_2_O_3_, and SiO_2_.

### Catalyst characterization

The BET surface area was measured on a Micromeritics 2020 with liquid nitrogen cooling. The samples were degassed under vacuum for 10 h, followed by isothermal adsorption and desorption of nitrogen at − 196 °C. For the γ-Al_2_O_3_ supported catalysts, the degas temperature was 350 °C, while for SiAl and SiO_2_ supported catalysts, the degas temperature was 300 °C. The pore size distribution from the adsorption isotherm was calculated by the BJH method. CO and H_2_ pulse chemisorption were performed at 35 °C with H_2_ or CO after reduction followed by flushing with UHP argon for 15 min using an ASAP 2920 (Micromeritics) instrument. A stoichiometric factor of 1 was used to calculate Pt, and a stoichiometric factor of 2 was used to calculate other metals dispersion [63].

### Reaction testing

In a catalytic bed, SiO_2_ beads and glass wools fixed 300 mg of the catalysts in a 1/4 inch Swagelok stainless tube. Before each run, the whole reactor setup was purged by a He flow and the packed catalysts were in-situ reduced with hydrogen for 2 h. After reduction, the flow gas was switched back to He and wait until catalytic bed equilibrated at 250 °C. The reactant, 1,8-cineole was injected into the preheater with a 1.0 ml/hour flow rate by a syringe pump. For the limonene tests, an equal molar flow rate was used. The preheater was set at 200 °C to fully evaporate the reactants. The evaporated reactants were mixed with a 20 mL/min He flow before introduction to the fixed-bed reactor. The fixed-bed reactor was held at 250 °C during the reaction. The outlet of the reactor connected to a water-cooled condenser to collect the products. The condensed products were collected every hour and analyzed by GC–MS/FID.

### Product characterization

The collected products were dissolved in ethanol and further analyzed by GC–MS. A DB-WAX column (30 m × 0.25 mm × 0.25 μm) from Agilent was applied for gas-chromatography analysis. The column temperature was increased by 10 °C/min from 50 °C to 230 °C and held at 230 °C for three minutes. The products were quantified using a flame ionization detector (FID) and 5795C mass spectrometry. The product composition represented the average over the time period. For example, the composition reported for the *t *= 1 h sample was the accumulated product from 0 to 1 h. The results were reported in mass selectivity, which is defined as,

1 $$\mathrm{Selectivity}=\frac{\mathrm{Mass\, of }\, p-\mathrm{Cymene}}{\mathrm{Collected\, mass\, in \,the \,condensor}}$$

In the limonene and 1,8-cineole in dodecane trials, the yield is defined as

2 $$\mathrm{Yield}=\frac{\mathrm{Mass \,of } \,p-\mathrm{Cymene x Mass\, ratio \,of \,limonene\, in\, reactant}}{\mathrm{Collected \,product\, mass \,in \,the \,condensor}}$$

### Fed batch cultivation for 1,8-cineole production

Fed-batch fermentation was performed in a 2-L bioreactor (Sartorius BIOSTAT B plus). A 5-mL culture was inoculated from a frozen glycerol stock, grown for 24 h, and then used to inoculate a 50-mL culture in a 250-mL flask; this culture was then used to inoculate the bioreactor to an OD_600nm_ of 0.1. The medium for batch phase (referred to as fermentation media) was M9 minimal medium supplemented with 2 mM MgSO_4_, 1 mg/L thiamine, 10 μM FeSO_4_, 0.1 mM CaCl_2_, 9.3 mM NH_4_Cl (when necessary), 1.5% glucose (w/v), 5 g/L yeast extract, 30 mg/L chloramphenicol, 100 mg/L carbenicillin, and micronutrients including 4 × 10^–6^ M boric acid, 3 × 10^–7^ M CoCl_2_, 1.5 × 10^–7^ M CuSO_4_, 3 × 10^–8^ M (NH_4_)_6_Mo_7_O_24_, 8 × 10^–7^ M MnCl_2_, and 1 × 10^–7^ M ZnSO_4_. The batch volume was 1 L, the pH of the culture was maintained at 7.0 using a base solution (10 N KOH), the temperature was set to 30 °C, the DO to 30%, airflow rate to 1 vvm (volume of air per volume of liquid per minute), stirring 600 rpm (then controlled by DO). Protein expression was induced with 1 mM IPTG when the culture reached an OD_600nm_ of 1–1.2 and dodecane (20% v/v) was also added. During the fed-batch phase, additional dodecane was added so its volume never decreased below 15% of the total volume. Feeding started when the initial amount of glucose was depleted (indicated by a sharp increase in DO or HPLC analysis) with a feed solution containing 200 g/L glucose, 15 g/L MgSO_4_ *7H_2_O, 5 g/L yeast extract, micronutrients according to previous descriptions (Korz et al., 1995) and appropriate antibiotics. Antifoam B (Sigma-Aldrich, St. Louis, MO) was added to the bioreactor when required. For exponential feeding, the feeding rate was changed every hour (for a total of 12 h) and calculated according to the following equation [64]:

3 $$m\left(t\right)=\left(\frac{\mu }{{Y}_{X/S}}+m\right){V}_{{t}_{F}}{X}_{{t}_{F}}{e}^{\mu \left(t-{t}_{F}\right)}$$

where *m(t)* is the mass flow of the substrate (g/h), *µ* is the specific growth rate (0.1 h^−1^), *Y*_*X/S*_ is the biomass/substrate yield coefficient (0.5 g/g), *m* is the specific maintained coefficient (0.025 g g^−1^ h^−1^), *V*_*tF*_ is the cultivation volume at the time of feeding (t_F_) and *X*_*tF*_ is the biomass concentration (g/L). After 12 h of exponential feeding, the feeding rate was maintained constant and glucose was continuously measured in the medium, and the feeding rate was adjusted to prevent its accumulation at more than 2 g/L. For pulse feeding based on DO, a fixed amount of feed solution was added to the tank and the DO of the culture was monitored, when the glucose was consumed (indicated by a sharp increase in DO) additional feed solution was added. The feed solution was added at a rate of 0.12–0.15 mL/min and the amount of feed solution added to the tank was adjusted based on the glucose consumption rate observed. Samples were taken from the dodecane overlay and 1,8-cineole was quantified as before, except for concentrations higher than 2 g/L the samples, where diluted 1:2; concentrations correspond to the amount of 1,8-cineole per volume of culture at the time of the sampling.

## Supplementary Information

**Additional file 1: Figure S1.** Nitrogen physisorption isotherms of Pd/SiAl. **Figure S2.** Nitrogen physisorption isotherms of Pd/SiO_2_. **Figure S3.** Nitrogen physisorption isotherms of Pd/Al_2_O_3_. **Figure S4.** Nitrogen physisorption isotherms of Al_2_O_3_. **Figure S5.** Nitrogen physisorption isotherms of Ni/Al_2_O_3_. **Figure S6.** Nitrogen physisorption isotherms of Cu/Al_2_O_3_. **Figure S7.** Nitrogen physisorption isotherms of Pt/Al_2_O_3_. **Figure S8.** Mass spectrum of limonene. **Figure S9.** Mass spectrum of 1,8-cineole. **Figure S10.** Mass spectrum of *p*-cymene. **Figure S11.** GC–MS spectrum of products of Pd/SiO_2_ catalytic reaction with limonene for 3 h. **Figure S12.** GC–MS spectrum of products of Pd/SiO_2_ catalytic reaction with 1,8-cineole for 3 h. **Figure S13.** GC–MS spectrum of products of γ-Al_2_O_3_ catalytic reaction with limonene for 3 h. **Figure S14.** GC–MS spectrum of products of γ-Al_2_O_3_ catalytic reaction with 1,8-cineole for 3 h. **Figure S15.** Deactivation of 100 mg of 5 wt% Pd/Al_2_O_3_ with (A) limonene (B)1,8-cineole as the reactants under 250 °C. **Figure S16.** Production of 1,8-cineole. (A) production at different C/N ratios; (B) production at different induction conditions. **Figure S17**. Production of 1,8-cineole with HMGR_Da expressed from different plasmid configurations. (A) Plasmid configurations (HMGR_Da in the plasmid is highlighted); (B) 1, 8-cineole titers; (C) Pyruvate accumulation. Production was done in test tubes using fermentation media supplemented with 1.5% glucose, grown at 30 °C. **Figure S18.** Fed-batch fermentation for Ferm 3_Da. **Figure S19.** Glucose consumption comparison for Ferm 2 and Ferm 3.

## Data Availability

Not applicable.

## References

[1] Wheeldon I, Christopher P, Blanch H (2017). Integration of heterogeneous and biochemical catalysis for production of fuels and chemicals from biomass. Curr Opin Biotechnol.

[2] Huo J, Shanks BH (2020). Bioprivileged molecules: integrating biological and chemical catalysis for biomass conversion. Annu Rev Chem Biomol Eng.

[3] Mendez-Perez D, Alonso-Gutierrez J, Hu QJ, Molinas M, Baidoo EEK, Wang G, Chan LJG, Adams PD, Petzold CJ, Keasling JD (2017). Production of jet fuel precursor monoterpenoids from engineered Escherichia coli. Biotechnol Bioeng.

[4] Lin YC, Huber GW (2009). The critical role of heterogeneous catalysis in lignocellulosic biomass conversion. Energy Environ Sci.

[5] Vennestrom PNR, Christensen CH, Pedersen S, Grunwaldt JD, Woodley JM (2010). Next-generation catalysis for renewables: combining enzymatic with inorganic heterogeneous catalysis for bulk chemical production. ChemCatChem.

[6] Schwartz TJ, Goodman SM, Osmundsen CM, Taarning E, Mozuch MD, Gaskell J, Cullen D, Kersten PJ, Dumesic JA (2013). Integration of chemical and biological catalysis: production of furylglycolic acid from glucose via cortalcerone. ACS Catal.

[7] Anbarasan P, Baer ZC, Sreekumar S, Gross E, Binder JB, Blanch HW, Clark DS, Toste FD (2012). Integration of chemical catalysis with extractive fermentation to produce fuels. Nature.

[8] Werpy T, Petersen G, Aden A, Bozell J, Holladay J, White J, Manheim M, Eliot D, Lasure L, Jones S (2004). Top value added chemicals from biomass. Volume 1-Results of screening for potential candidates from sugars and synthesis gas. Technical Report..

[9] Bozell JJ, Petersen GR (2010). Technology development for the production of biobased products from biorefinery carbohydrates-the US Department of Energy's "Top 10" revisited. Green Chem.

[10] Shanks BH, Keeling PL (2017). Bioprivileged molecules: creating value from biomass. Green Chem.

[11] Wojcieszak R, Santarelli F, Paul S, Dumeignil F, Cavani F, Gonçalves RV (2015). Recent developments in maleic acid synthesis from bio-based chemicals. Sustainable Chem Processes.

[12] Osswald P, Whitside R, Schaffer J, Kohler M (2017). An experimental flow reactor study of the combustion kinetics, of terpenoid jet fuel compounds: Farnesane, p-menthane and p-cymene. Fuel.

[13] Hu PH, Tan MX, Cheng L, Zhao HY, Feng R, Gu WJ, Han W (2019). Bio-inspired iron-catalyzed oxidation of alkylarenes enables late-stage oxidation of complex methylarenes to arylaldehydes. Nature Commun.

[14] Yu H, Ru S, Dai GY, Zhai YY, Lin HL, Han S, Wei YG (2017). An Efficient iron(III)-catalyzed aerobic oxidation of aldehydes in water for the green preparation of carboxylic acids. Angew Chem Intern Edition.

[15] Nakamura R, Obora Y, Ishii Y (2009). Synthesis of 6-hydroxy-2-naphthoic acid from 2,6-diisopropylnaphthalene using NHPI as a key catalyst. Tetrahedron.

[16] Colonna M, Berti C, Fiorini M, Binassi E, Mazzacurati M, Vannini M, Karanam S (2011). Synthesis and radiocarbon evidence of terephthalate polyesters completely prepared from renewable resources. Green Chem.

[17] Wichterlova B, Cejka J (1994). Mechanism of n-propyltoluene formation in c3 alkylation of toluene—the effect of zeolite structural type. J Catal.

[18] Yu CC, Tan CS (2007). Production of para-cymene from alkylation of toluene with propylene in supercritical CO(2) over shape-selective HZSM-5 pellets. Ind Eng Chem Res.

[19] Tholl D (2015). Biotechnology of Isoprenoids. Adv Biochem Eng Biotechnol.

[20] Jongedijk E, Cankar K, Buchhaupt M, Schrader J, Bouwmeester H, Beekwilder J (2016). Biotechnological production of limonene in microorganisms. Appl Microbiol Biotechnol.

[21] Ciriminna R, Lomeli-Rodriguez M, Cara PD, Lopez-Sanchez JA, Pagliaro M (2014). Limonene: a versatile chemical of the bioeconomy. Chem Commun.

[22] Martin-Luengo MA, Yates M, Domingo MJM, Casal B, Iglesias M, Esteban M, Ruiz-Hitzky E (2008). Synthesis of p-cymene from limonene, a renewable feedstock. Appl Catal B-Environ.

[23] Makarouni D, Lycourghiotis S, Kordouli E, Bourikas K, Kordulis C, Dourtoglou V (2018). Transformation of limonene into p-cymene over acid activated natural mordenite utilizing atmospheric oxygen as a green oxidant: A novel mechanism. Appl Catal B-Environ.

[24] Beller HR, Lee TS, Katz L (2015). Natural products as biofuels and bio-based chemicals: fatty acids and isoprenoids. Nat Prod Rep.

[25] Martin-Luengo MA, Yates M, Rojo ES, Arribas DH, Aguilar D, Hitzky ER (2010). Sustainable p-cymene and hydrogen from limonene. Appl Catal A-Gen.

[26] Kamitsou M, Panagiotou GD, Triantafyllidis KS, Bourikas K, Lycourghiotis A, Kordulis C (2014). Transformation of alpha-limonene into p-cymene over oxide catalysts: a green chemistry approach. Appl Catal A-Gen.

[27] Fernandes C, Catrinescu C, Castilho P, Russo PA, Carrott MR, Breen C (2007). Catalytic conversion of limonene over acid activated serra de dentro (SD) bentonite. Appl Catal A-Gen.

[28] Palmer RC (1942). Developments in terpene chemicals. Ind Eng Chem.

[29] Lesage P, Candy JP, Hirigoyen C, Humblot F, Basset JM (1996). Selective dehydrogenation of dipentene (R-(+)-limonene) into paracymene on silica supported palladium assisted by alpha-olefins as hydrogen acceptor. J Mol Catal A-Chem.

[30] Weyrich PA, Holderich WF (1997). Dehydrogenation of alpha-limonene over Ce promoted, zeolite supported Pd catalysts. Appl Catal A-Gen.

[31] Grau RJ, Zgolicz PD, Gutierrez C, Taher HA (1999). Liquid phase hydrogenation, isomerization and dehydrogenation of limonene and derivatives with supported palladium catalysts. J Mol Catal A-Chem.

[32] Buhl D, Roberge DM, Holderich WF (1999). Production of p-cymene from alpha-limonene over silica supported Pd catalysts. Appl Catal A-Gen.

[33] Buhl D, Weyrich PA, Sachtler WMH, Holderich WF (1998). Support effects in the Pd catalyzed dehydrogenation of terpene mixtures to p-cymene. Appl Catal A-Gen.

[34] Zhao C, Gan W, Fan XB, Cai ZP, Dyson PJ, Kou Y (2008). Aqueous-phase biphasic dehydroaromatization of bio-derived limonene into p-cymene by soluble Pd nanocluster catalysts. J Catal.

[35] Zhang QG, Bi LW, Zhao ZD, Chen YP, Li DM, Gu Y, Wang J, Chen YX, Bo CY, Liu XZ (2010). Application of ultrasonic spraying in preparation of p-cymene by industrial dipentene dehydrogenation. Chem Eng J.

[36] Zhang JJ, Zhao C (2015). A new approach for bio-jet fuel generation from palm oil and limonene in the absence of hydrogen. Chem Commun.

[37] Zhang JJ, Zhao C (2016). Development of a bimetallic Pd-Ni/HZSM-5 catalyst for the tandem limonene dehydrogenation and fatty acid deoxygenation to alkanes and arenes for use as biojet fuel. ACS Catal.

[38] Yilmazoglu E, Akgun M (2018). p-Cym. ene production from orange peel oil using some metal catalyst in supercritical alcohols. J Supercrit Fluids.

[39] Leita BA, Warden AC, Burke N, O'Shea MS, Trimm D (2010). Production of p-cymene and hydrogen from a bio-renewable feedstock-1,8-cineole (eucalyptus oil). Green Chem.

[40] Leita BA, Gray P, O'Shea M, Burke N, Chiang K, Trimm D (2011). The conversion of 1,8-cineole sourced from renewable Eucalyptus oil to p-cymene over a palladium doped gamma-Al2O3 catalyst. Catal Today.

[41] Alonso-Gutierrez J, Chan R, Batth TS, Adams PD, Keasling JD, Petzold CJ, Lee TS (2013). Metabolic engineering of Escherichia coli for limonene and perillyl alcohol production. Metab Eng.

[42] Wang X, Pereira JH, Tsutakawa S, Fang XY, Adams PD, Mukhopadhyay A, Lee TS (2021). Efficient production of oxidized terpenoids via engineering fusion proteins of terpene synthase and cytochrome P450. Metab Eng.

[43] Alonso-Gutierrez J, Kim EM, Batth TS, Cho N, Hu QJ, Chan LJG, Petzold CJ, Hinson NJ, Adams PD, Keasling JD (2015). Principal component analysis of proteomics (PCAP) as a tool to direct metabolic engineering. Metab Eng.

[44] Peralta-Yahya PP, Ouellet M, Chan R, Mukhopadhyay A, Keasling JD, Lee TS (2011). Identification and microbial production of a terpene-based advanced biofuel. Nature Commun.

[45] Kang A, George KW, Wang G, Baidoo E, Keasling JD, Lee TS (2016). Isopentenyl diphosphate (IPP)-bypass mevalonate pathways for isopentenol production. Metab Eng.

[46] Ren YY, Liu SS, Jin GJ, Yang XB, Zhou YJJ (2020). Microbial production of limonene and its derivatives: achievements and perspectives. Biotechnol Adv.

[47] Wen GD, Xu YP, Ma HJ, Xu ZS, Tian ZJ (2008). Production of hydrogen by aqueous-phase reforming of glycerol. Int J Hydrog Energy.

[48] Chacon MG, Marriott A, Kendrick EG, Styles MQ, Leak DJ (2019). Esterification of geraniol as a strategy for increasing product titre and specificity in engineered Escherichia coli. Microb Cell Fact.

[49] Enjalbert B, Millard P, Dinclaux M, Portais JC, Letisse F (2017). Acetate fluxes in Escherichia coli are determined by the thermodynamic control of the Pta-AckA pathway. Sci Rep.

[50] Kotte O, Volkmer B, Radzikowski JL, Heinemann M (2014). Phenotypic bistability in Escherichia coli's central carbon metabolism. Mol Syst Biol.

[51] Kang A, Mendez-Perez D, Goh EB, Baidoo EEK, Benites VT, Beller HR, Keasling JD, Adams PD, Mukhopadhyay A, Lee TS (2019). Optimization of the IPP-bypass mevalonate pathway and fed-batch fermentation for the production of isoprenol in Escherichia coli. Metab Eng.

[52] De Mey M, De Maeseneire S, Soetaert W, Vandamme E (2007). Minimizing acetate formation in E-coli fermentations. J Ind Microbiol Biotechnol.

[53] Eiteman MA, Altman E (2006). Overcoming acetate in Escherichia coli recombinant protein fermentations. Trends Biotechnol.

[54] Clark DP (1989). The fermentation pathways of Escherichia-coli. FEMS Microbiol Lett.

[55] Chang DE, Shin S, Rhee JS, Pan JG (1999). Acetate metabolism in a pta mutant of Escherichia coli W3110: Importance of maintaining acetyl coenzyme a flux for growth and survival. J Bacteriol.

[56] de Graef MR, Alexeeva S, Snoep JL, de Mattos MJT (1999). The steady-state internal redox state (NADH/NAD) reflects the external redox state and is correlated with catabolic adaptation in Escherichia coli. J Bacteriol.

[57] Cassey B, Guest JR, Attwood MM (1998). Environmental control of pyruvate dehydrogenase complex expression in Escherichia coli. FEMS Microbiol Lett.

[58] Snoep JL, Degraef MR, Westphal AH, Dekok A, Demattos MJT, Neijssel OM (1993). Differences in sensitivity to nadh of purified pyruvate-dehydrogenase complexes of enterococcus-faecalis, lactococcus-lactis, azotobacter-vinelandii and Escherichia-coli—implications for their activity in-vivo. FEMS Microbiol Lett.

[59] Dugar D, Stephanopoulos G (2011). Relative potential of biosynthetic pathways for biofuels and bio-based products. Nat Biotechnol.

[60] Ma SM, Garcia DE, Redding-Johanson AM, Friedland GD, Chan R, Batth TS, Haliburton JR, Chivian D, Keasling JD, Petzold CJ (2011). Optimization of a heterologous mevalonate pathway through the use of variant HMG-CoA reductases. Metab Eng.

[61] Kirby J, Geiselman GM, Yaegashi J, Kim J, Zhuang X, Tran-Gyamfi MB, Prahl JP, Sundstrom ER, Gao YQ, Munoz N (2021). Further engineering of R toruloides for the production of terpenes from lignocellulosic biomass. Biotechnol Biofuels..

[62] Lambert RJW, Pearson J (2000). Susceptibility testing: accurate and reproducible minimum inhibitory concentration (MIC) and non-inhibitory concentration (NIC) values. J Appl Microbiol.

[63] Huo JJ, Duan P, Pham HN, Chan YJ, Datye AK, Schmidt-Rohr K, Shanks BH (2018). Improved hydrothermal stability of Pd nanoparticles on nitrogen-doped carbon supports. Catal Sci Technol.

[64] Korz DJ, Rinas U, Hellmuth K, Sanders EA, Deckwer WD (1995). Simple fed-batch technique for high cell-density cultivation of escherichia-coli. J Biotechnol.

