# Supplementary material for: Precursor prioritization for p-cymene production through synergistic integration of biology and chemistry
Source: Biotechnol Biofuels Bioprod. 2022 Nov 17;15:126. doi: 10.1186/s13068-022-02226-7 (PMC9670573; doi:10.1186/s13068-022-02226-7)
Supplement: Supplementary file 1 — Additional file 1: Figure S1. Nitrogen physisorption isotherms of Pd/SiAl. Figure S2. Nitrogen physisorption isotherms of Pd/SiO2. Figure S3. Nitrogen physisorption isotherms of Pd/Al2O3. Figure S4. Nitrogen physisorption isotherms of Al2O3. Figure S5. Nitrogen physisorption isotherms of Ni/Al2O3. Figure S6. Nitrogen physisorption isotherms of Cu/Al2O3. Figure S7. Nitrogen physisorption isotherms of Pt/Al2O3. Figure S8. Mass spectrum of limonene. Figure S9. Mass spectrum of 1,8-cineole. Figure S10. Mass spectrum of p-cymene. Figure S11. GC–MS spectrum of products of Pd/SiO2 catalytic reaction with limonene for 3 h. Figure S12. GC–MS spectrum of products of Pd/SiO2 catalytic reaction with 1,8-cineole for 3 h. Figure S13. GC–MS spectrum of products of γ-Al2O3 catalytic reaction with limonene for 3 h. Figure S14. GC–MS spectrum of products of γ-Al2O3 catalytic reaction with 1,8-cineole for 3 h. Figure S15. Deactivation of 100 mg of 5 wt% Pd/Al2O3 with (A) limonene (B)1,8-cineole as the reactants under 250 °C. Figure S16. Production of 1,8-cineole. (A) production at different C/N ratios; (B) production at different induction conditions. Figure S17. Production of 1,8-cineole with HMGR_Da expressed from different plasmid configurations. (A) Plasmid configurations (HMGR_Da in the plasmid is highlighted); (B) 1, 8-cineole titers; (C) Pyruvate accumulation. Production was done in test tubes using fermentation media supplemented with 1.5% glucose, grown at 30 °C. Figure S18. Fed-batch fermentation for Ferm 3_Da. Figure S19. Glucose consumption comparison for Ferm 2 and Ferm 3. [file 13068_2022_2226_MOESM1_ESM.docx]

**Supplementary Information for “Precursor Prioritization for *p*-Cymene Production through Synergistic Integration of Biology and Chemistry”**

*Hsi-Hsin Lin,* ^1^*^,^*^2^*^,^*^3,§^ *Daniel Mendez‐Perez,* ^3^*^,^*^4,§^ *Jimin Park,* ^3^*^,^*^5^ *Xi Wang,* ^3^*^,^*^4^ *Yan Cheng,* ^1^*^,^*^2^ *Jiajie Huo,* ^1^*^,^*^2^ *Aindrila Mukhopadhyay,* ^3^*^,^*^4^ *Taek Soon Lee *^,^*^3^*^,^*^4^ *and Brent H. Shanks *^,^*^1^*^,^*^2^*^,^*^3^

^1^*Department of Chemical and Biological Engineering, Iowa State University, Ames, Iowa 50011, United States.*

^2^*NSF Engineering Research Center for Biorenewable Chemicals (CBiRC), Iowa State University, Ames, Iowa 50011, United States.*

^3^*Joint BioEnergy Institute, 5885 Hollis Street, Emeryville, CA 94608, United States.*

^4^*Biological Systems & Engineering Division, Lawrence Berkeley National Laboratory, Berkeley, CA, 94720, United States.*

^5^*Department of Chemical and Biomolecular Engineering, University of California, Berkeley, CA 94720, USA.*

1. **Nitrogen Physisorption Isotherms of Catalysts**

**
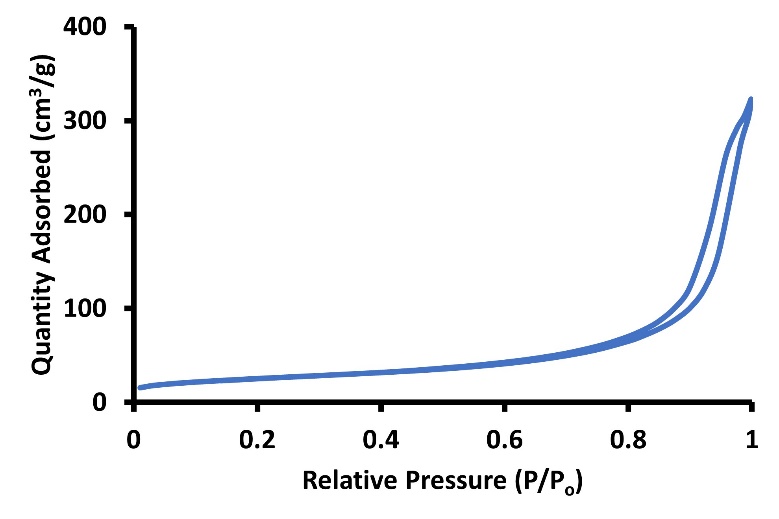
**

**Figure S1.** Nitrogen physisorption isotherms of Pd/SiAl.


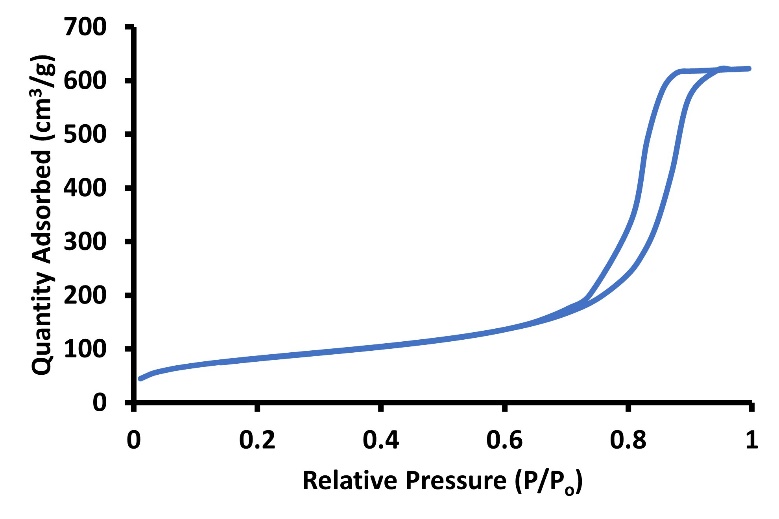


**Figure S2.** Nitrogen physisorption isotherms of Pd/SiO_2_.


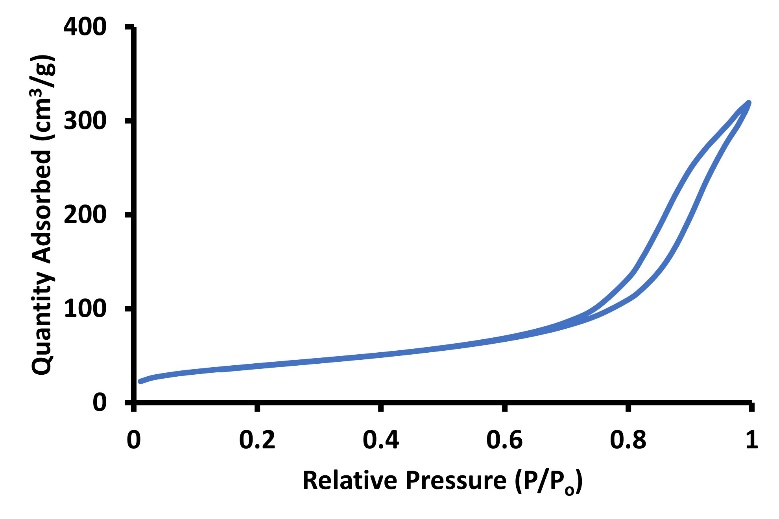


**Figure S3.** Nitrogen physisorption isotherms of Pd/Al_2_O_3_.


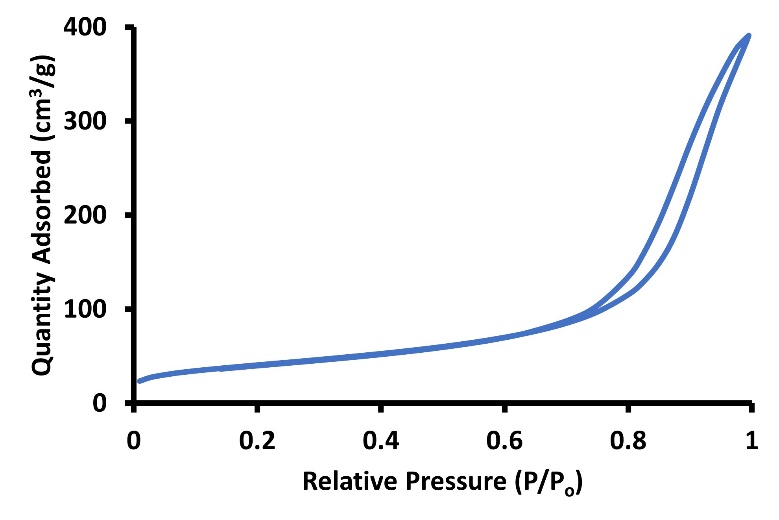


**Figure S4.** Nitrogen physisorption isotherms of Al_2_O_3_.


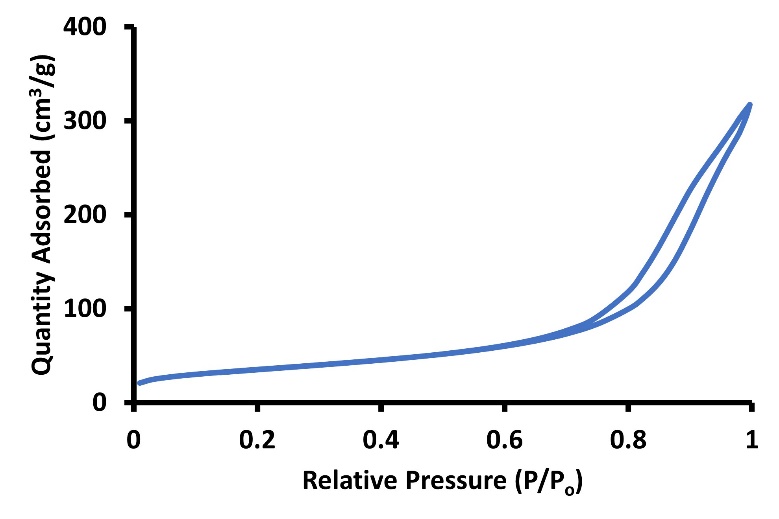


**Figure S5.** Nitrogen physisorption isotherms of Ni/Al_2_O_3_.


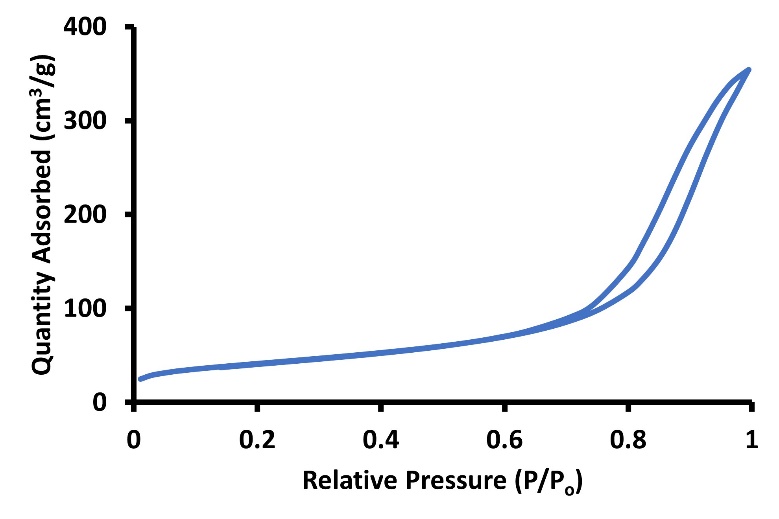


**Figure S6.** Nitrogen physisorption isotherms of Cu/Al_2_O_3_.


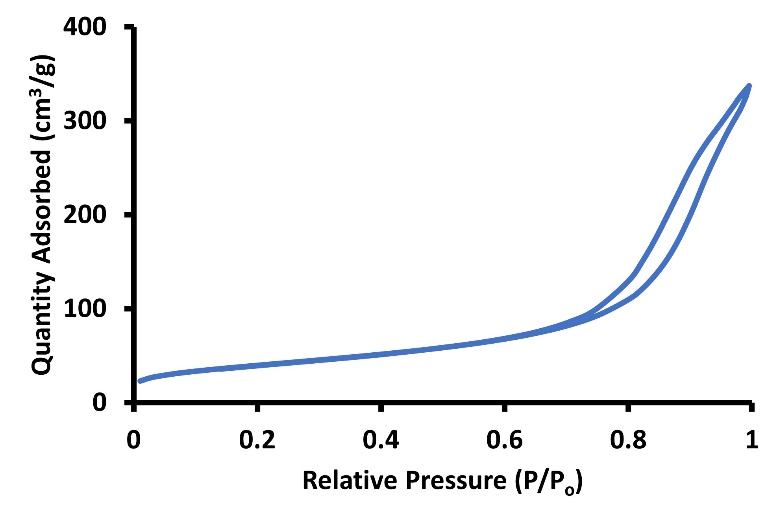


**Figure S7.** Nitrogen physisorption isotherms of Pt/Al_2_O_3_.

1. **Product GC-MS Analysis**


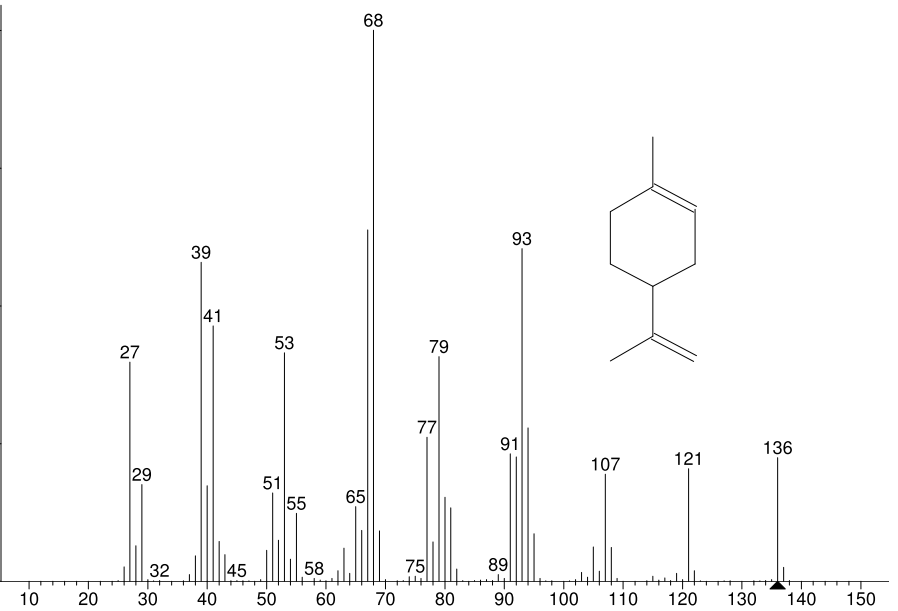


**Figure S8.** Mass spectrum of limonene.


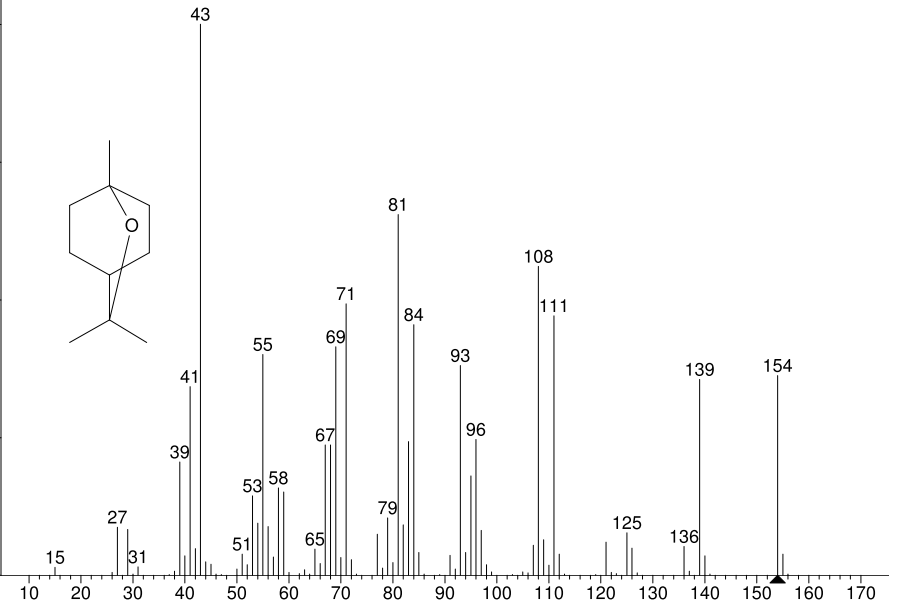


**Figure S9.** Mass spectrum of 1,8-cineole.


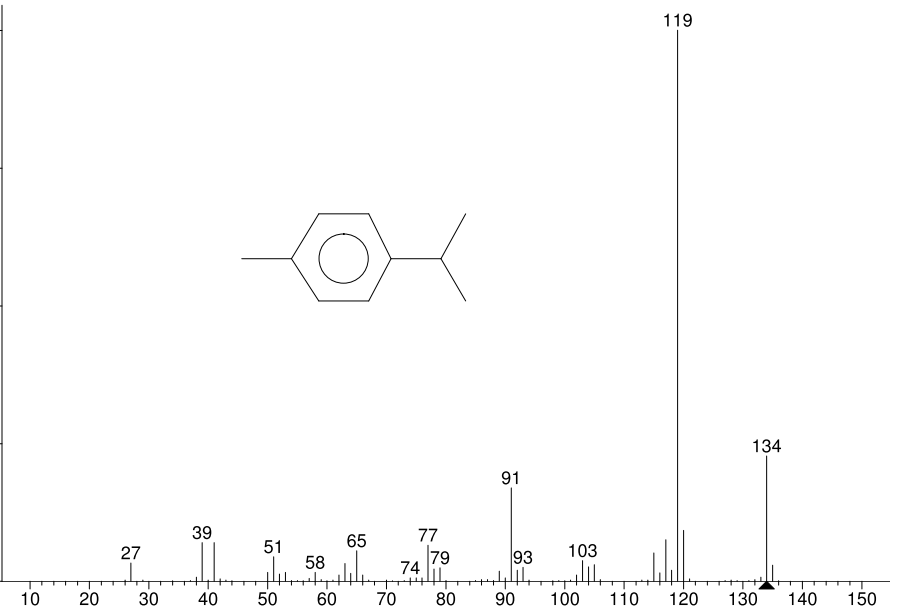


**Figure S10.** Mass spectrum of *p*-cymene.


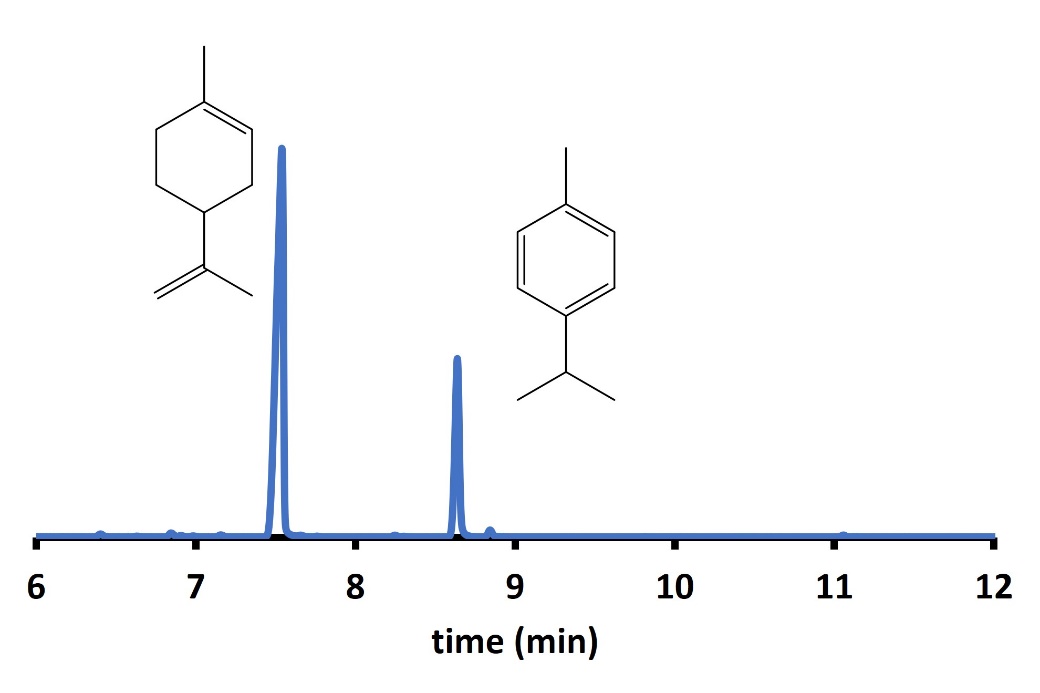


**Figure S11.** GC-MS spectrum of products of Pd/SiO_2_ catalytic reaction with limonene for 3 hours.


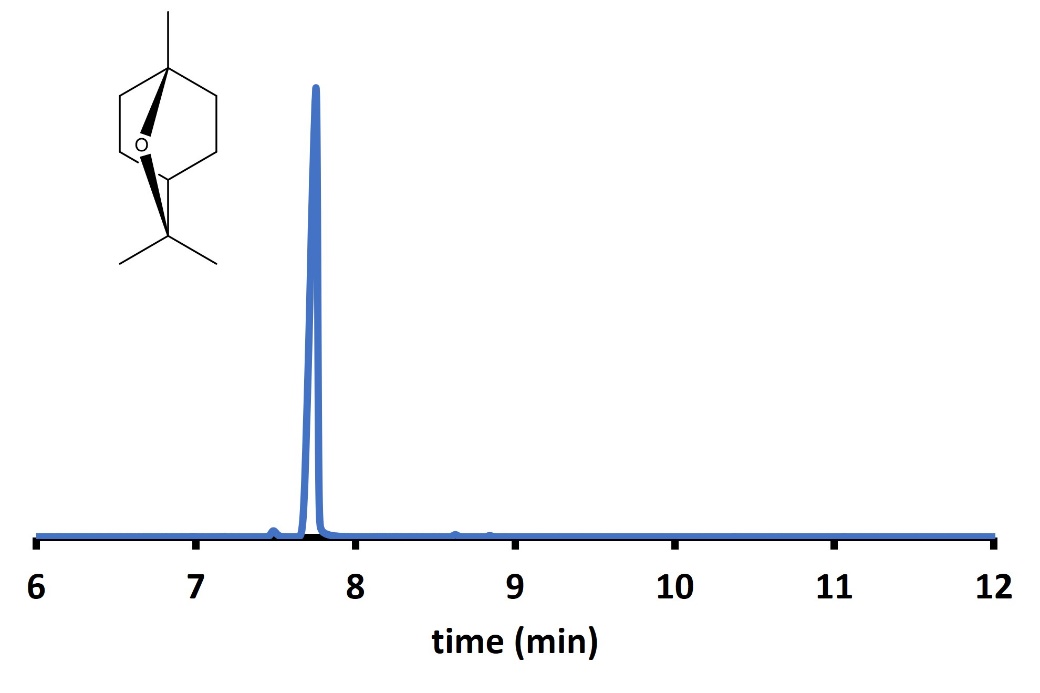


**Figure S12.** GC-MS spectrum of products of Pd/SiO_2_ catalytic reaction with 1,8-cineole for 3 hours.


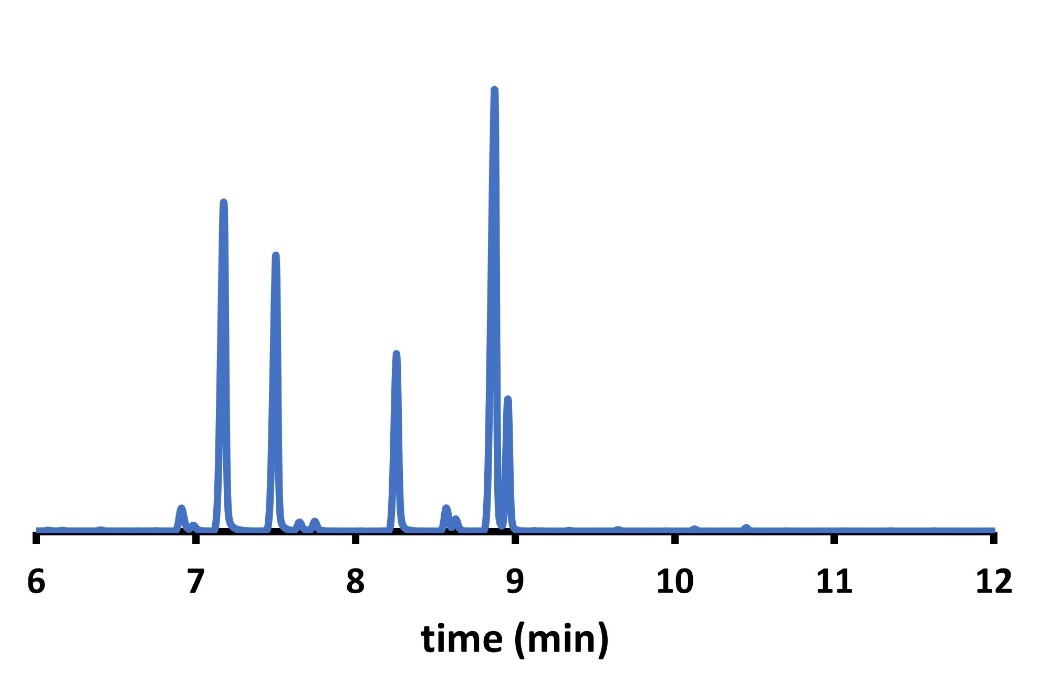


**Figure S13.** GC-MS spectrum of products of γ-Al_2_O_3_ catalytic reaction with limonene for 3 hours.


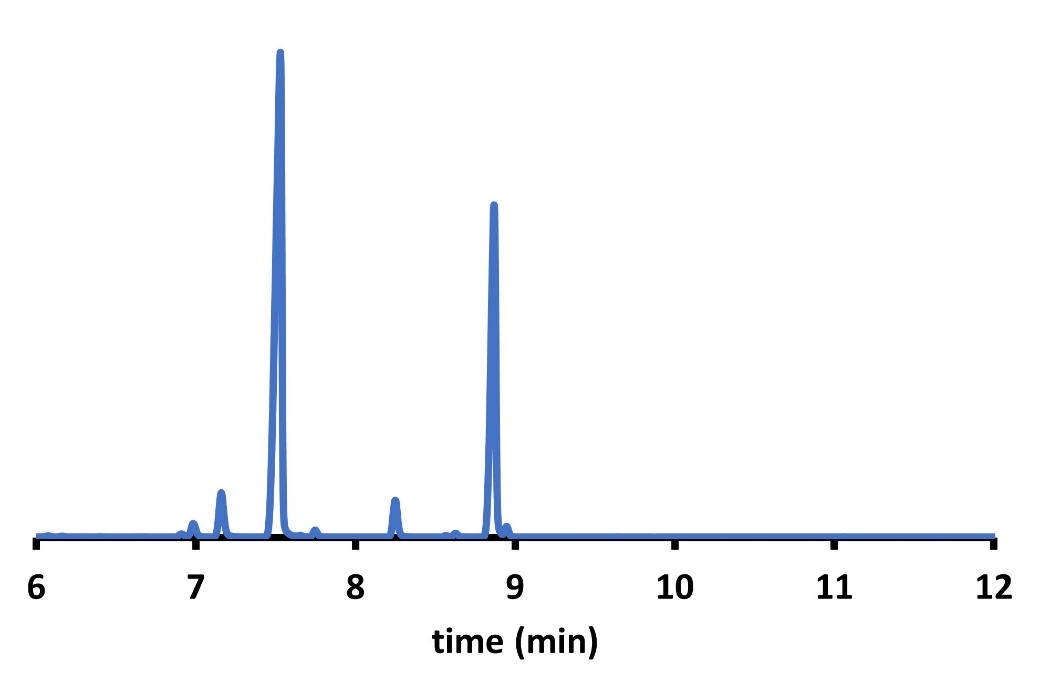


**Figure S14.** GC-MS spectrum of products of γ-Al_2_O_3_ catalytic reaction with 1,8-cineole for 3 hours.

1. **Catalysts Deactivation Experiment (100 mg)**

The Pd/Al_2_O_3_ and Pd/SiAl catalysts showed relatively stable yields with 300 mg of loading in 5 hours. Since the limonene and 1,8-cineole were nearly fully converted in these trials, the collected yields can be attributed to the high reactivity of the catalysts. Even the rapid catalyst deactivation occurs during the reaction under full conversion, remained active sites were still sufficient to convert the reactants. To study if the case above applied in our system, the catalysts loading in the catalytic bed were reduced to 100 mg. In Figure S1, deactivation of both catalysts was observed which confirmed the assumption we proposed in the previous section that catalysts deactivated continuously in all cases. The reason that reactivity remained unchanged was because of that only part of the catalysts was still active, but it was sufficient for the injected reactant. With further deactivation, the overall selectivity started decreasing when the remained active catalysts were insufficient.


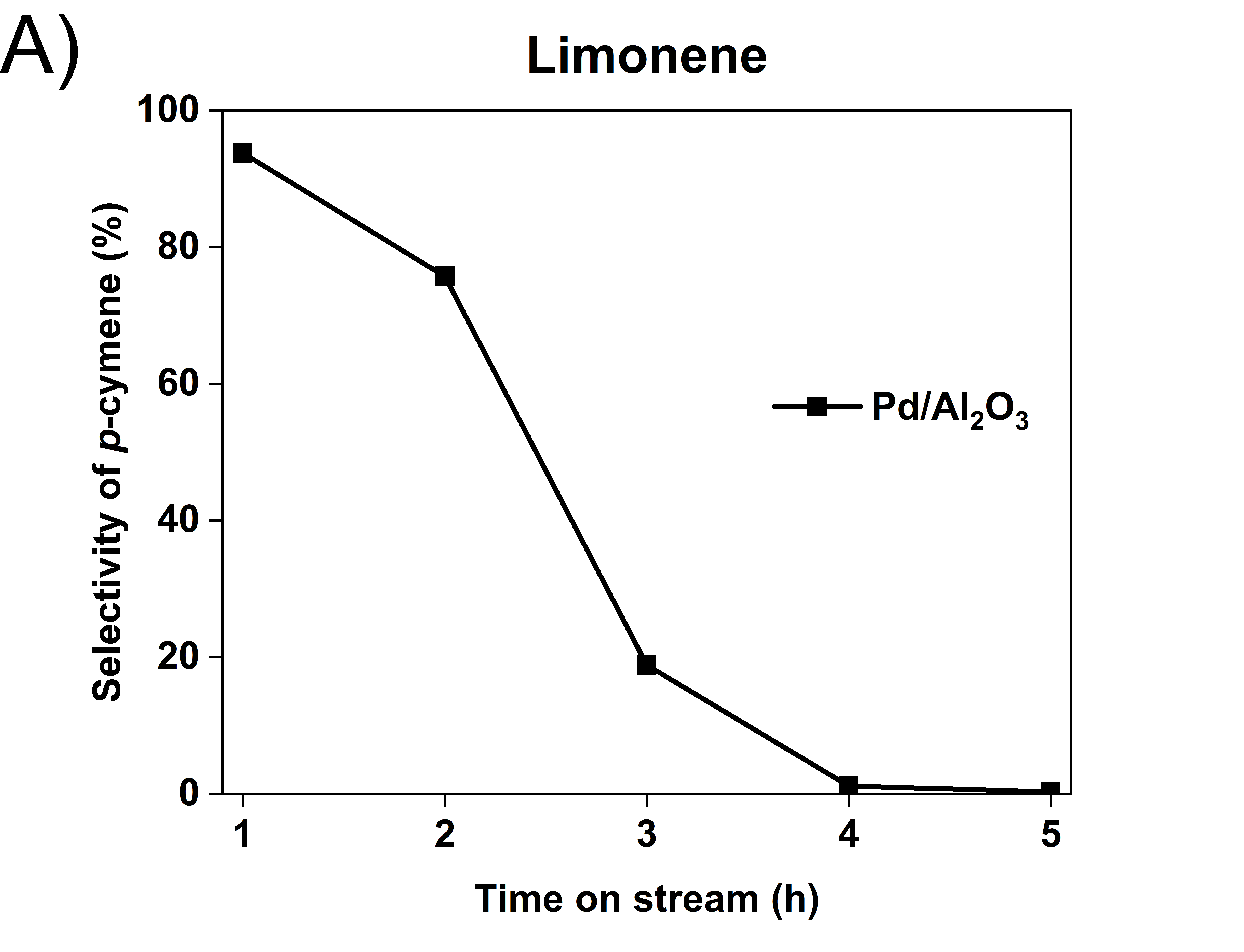

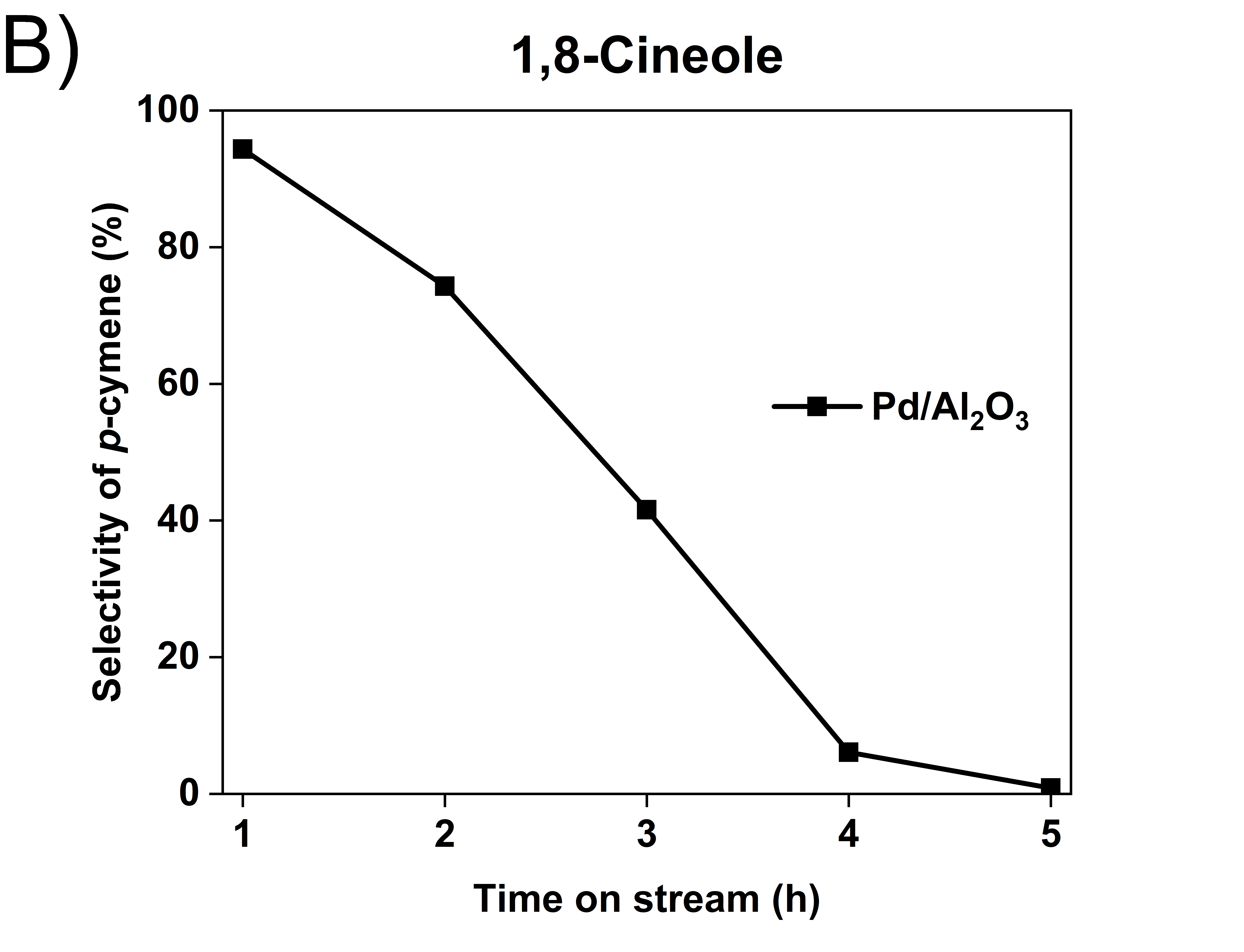


**Figure S15.** Deactivation of 100 mg of 5 wt% Pd/Al_2_O_3_ with (A) limonene (B)1,8-cineole as the reactants under 250^o^C.

1. **1,8-Cineole fermentation**


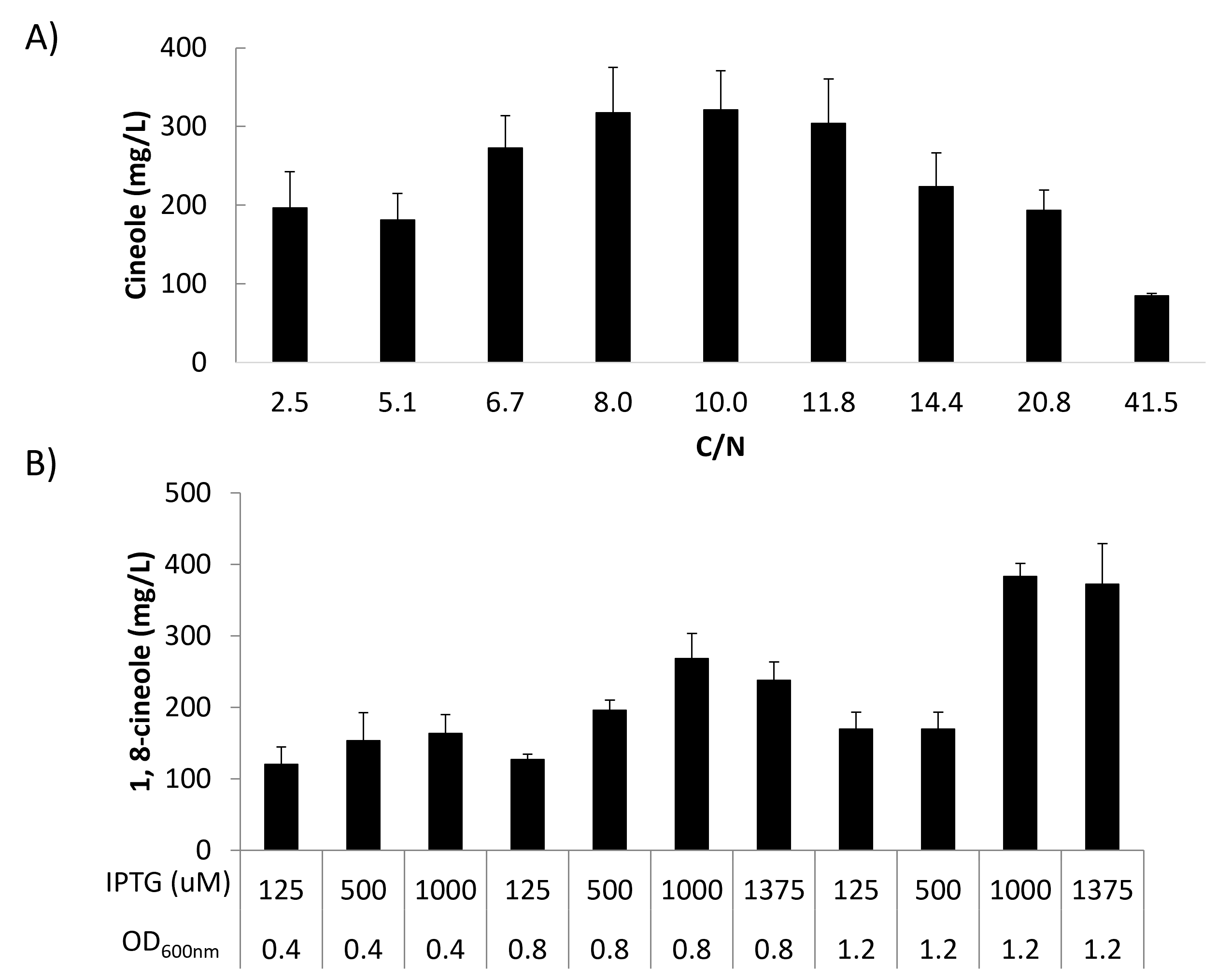


**Figure S16**. Production of 1,8-cineole. (A) production at different C/N ratios; (B) production at different induction conditions.


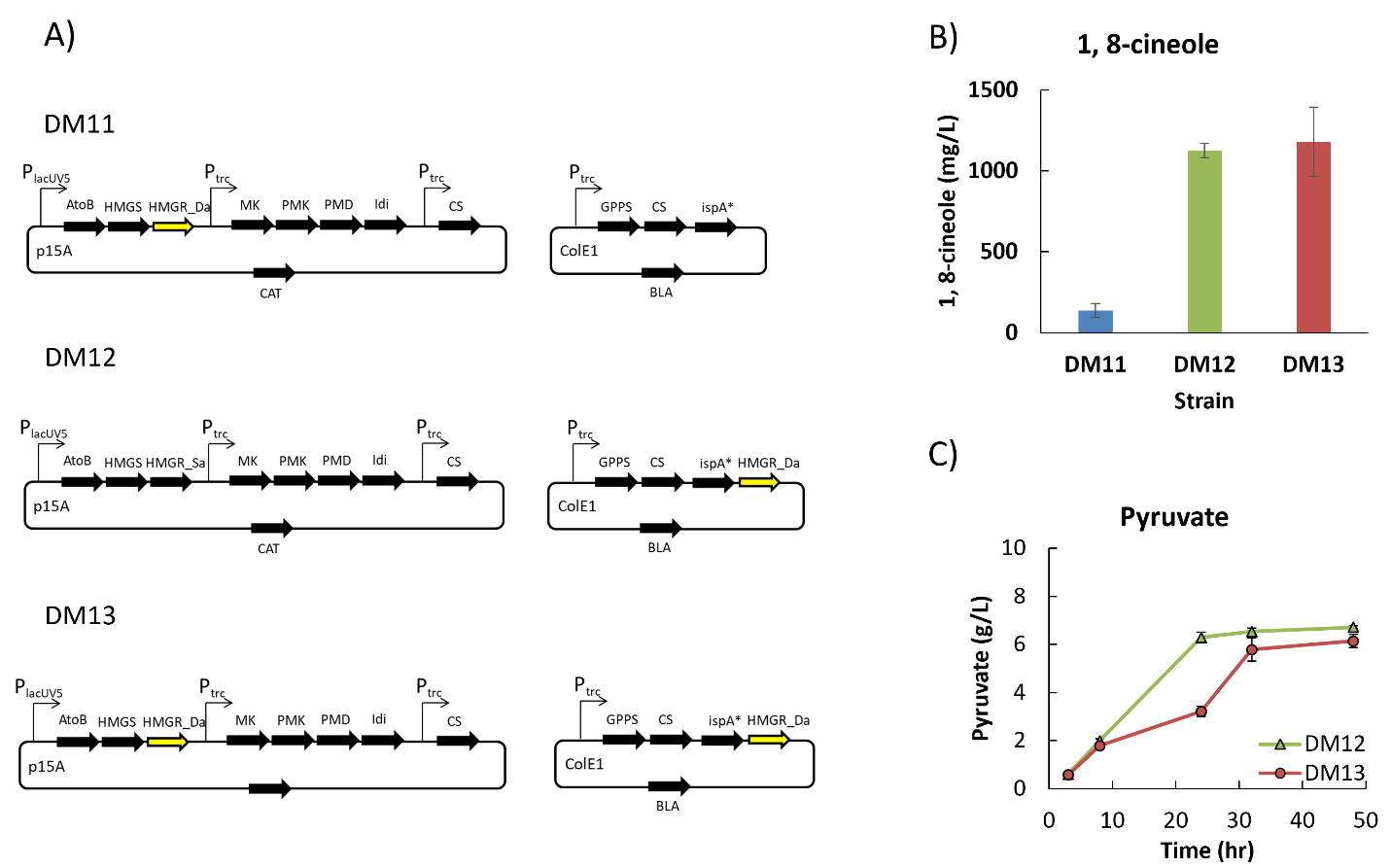


**Figure S17**. Production of 1,8- cineole with HMGR_Da expressed from different plasmid configurations. (A) Plasmid configurations (HMGR_Da in the plasmid is highlighted); (B) 1, 8-cineole titers; (C) Pyruvate accumulation. Production was done in test tubes using fermentation media supplemented with 1.5% glucose, grown at 30°C


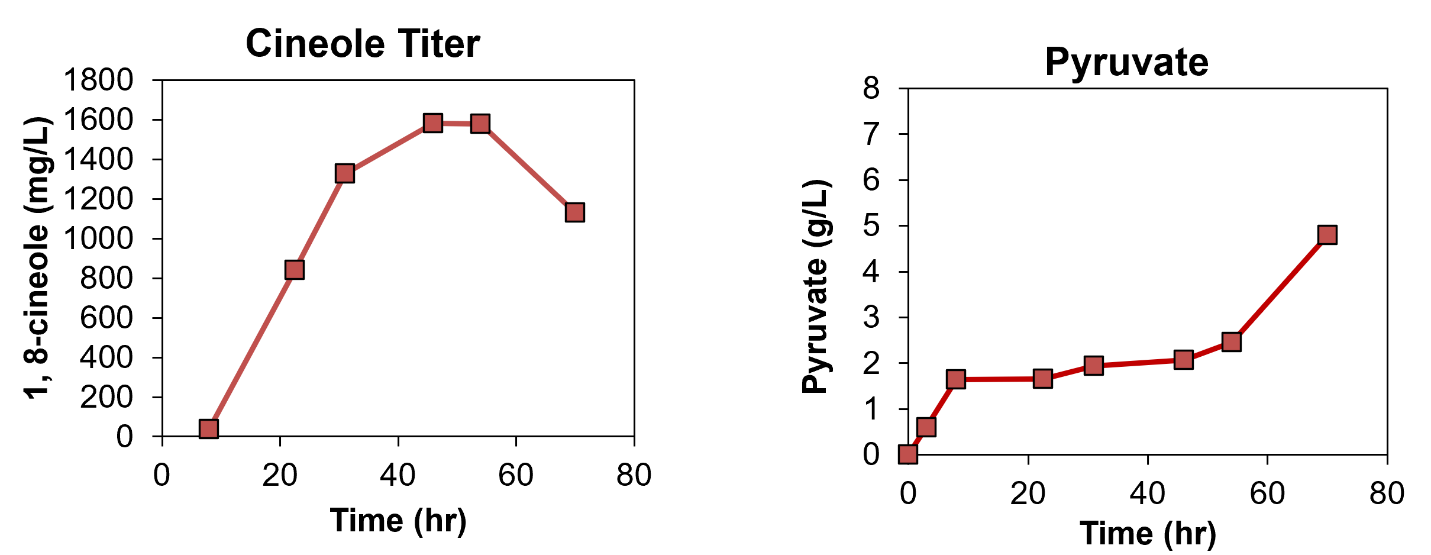


**Figure S18**. Fed-batch fermentation for Ferm 3_Da


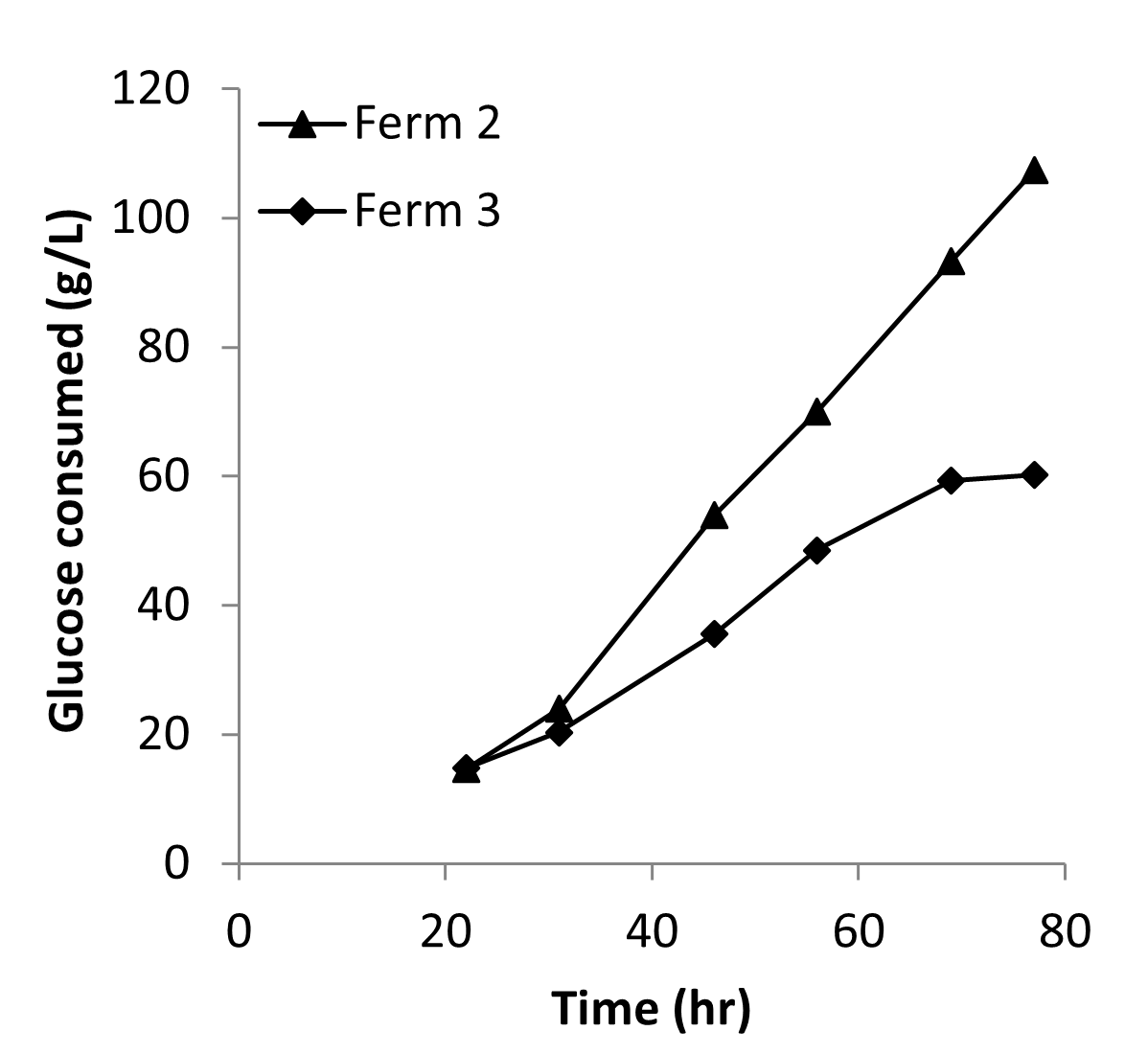


**Figure S19**. Glucose consumption comparison for Ferm 2 and Ferm 3
